# Short-term effectiveness of face-to-face periodic occupational health screening versus electronic screening with targeted follow-up: results from a quasi-randomized controlled trial in four Belgian hospitals

**DOI:** 10.5271/sjweh.4011

**Published:** 2022-03-31

**Authors:** Jonas Stefaan Steel, Lode Godderis, Jeroen Luyten

**Affiliations:** 1 Leuven Institute for Healthcare Policy, Department of Public Health and Primary Care, KU Leuven, Leuven, Belgium; 2 Environment and Health, Department of Public Health and Primary Care, KU Leuven, Leuven, Belgium; 3 IDEWE, External Service for Prevention and Protection at Work, Haasrode, Belgium

**Keywords:** health assessment, medical examination, mixed-effect model, multilevel modelling, occupational stressor, randomized controlled experiment

## Abstract

**Objectives:**

In many countries, organisations are legally obliged to have occupational physicians screen employees regularly. However, this system is time-intensive, and there may be more cost-effective alternatives. Our objective is to compare the short-term effectiveness of periodic occupational health screening of hospital employees by an occupational physician with a system of electronic screening with targeted follow-up.

**Methods:**

A randomized controlled trial was set up among personnel of four Belgian hospitals, with three measurement moments between June 2019 and December 2020, to compare differences in self-assessed health, healthcare use, productivity and intermediate outcomes over 19 months. Mixed effects models were used to assess differences in effectiveness. Superiority and non-inferiority post-hoc tests were used as a robustness check. The experiment coincided with the first two COVID-19 waves during which hospital employees were exposed to an exceptional period of occupational stress.

**Results:**

In total, 1077 employees (34% of the target population) participated. Although we observed some immediate effects of the intervention (less trust in the physician, absenteeism, and healthcare use), all these effects disappeared over time. After 19 months, including two waves of COVID-19 hospitalizations, no significant differences were observed between employees screened through face-to-face contact and those screened electronically.

**Conclusions:**

Our study finds no indication that, in the short-term, substituting physician screening of the workforce with a quicker survey-based screening with targeted follow-up has different effects on the studied endpoints. However, as health and disease are often the result of a long-term process, more evidence is needed to determine long-term effects.

Work and health are intricately linked. On the one hand, evidence points out that work is an important source of value and meaning (1, 2) and has positive effects on health (3, 4). On the other hand, the workplace can be a hazardous environment when employees are confronted with physical, chemical, biological, or psychosocial hazards (5, 6).

To minimize the impact of occupational risks on health and functioning, many countries make use of periodic occupational health screenings. In the US, the Occupational Safety and Health administration (OSHA) requires medical screening for numerous hazardous substances (7). In the EU (and UK), these routine medical examinations are offered to nearly all workers on an annual basis as an implementation of Article 14 of Directive 89/391/EEC (8, 9). The European Agency for Safety and Health at Work (EU-OSHA) survey among European workplaces – the European Survey of Enterprises on New and Emerging Risks (ESENER) – indicated that 65% of establishments (58% for micro and 89% for large enterprises) arrange regular medical examinations to monitor the health of employees (10). These periodic health screenings can include measuring biometrics and offer a clinical investigation by an occupational physician (9, 10). They focus on primary (eg, vaccination), secondary (eg, screening for diseases), and tertiary prevention (eg, return to work), often take a job-specific risk analysis as a starting point, and can lead to both individual (employee) and collective (worksite) measures (11, 12).

However, there is – as of yet – insufficient evidence on the effectiveness of health examinations, and more research is needed in this field. A systematic review of general health screening found 14 studies of sufficient quality, and did not find studies that indicated an effect on all-cause, cardiovascular, or cancer mortality. In contrast, it suggested they could lead to adverse effects such as overtreatment, misplaced reassurance, or (in the case of false positives) unnecessary worries about health (11). However, these health checks were not performed in an occupational health setting, and are therefore not readily transferable. Even if only high-risk individuals are targeted, it remains an open question whether their screening is beneficial (12), and more research is needed.

Another common challenge of such workers’ health surveillance systems is that they are demanding in terms of working hours. In Belgium for instance, an occupational physician sees about 70% of employees regularly, thus taking up a significant portion of both employees’ and physicians’ time (13). Meanwhile, occupational physicians are proving ever more difficult to recruit (14–16). Therefore, the question rises whether it is possible to allow for alternative, less labor-intensive means of screening workers without unduly compromising effectiveness.

A targeted approach might be more promising. A possible alternative is therefore to use a screening survey that selectively refers employees to the occupational physician when it detects indications of functioning and/or (work-related) health problems. This could save occupational physicians’ time, and it is *a priori* an attractive option for employers and employees. The former might save on screening costs, the latter might prefer a survey’s practical ease (an online survey can be completed everywhere). Research also points out that some health problems are more easily disclosed in surveys than during face-to-face consultations (17). Several countries already use surveys as surveillance systems to monitor occupational health (18–20), while others (eg, Finland and The Netherlands) have used health risk appraisals for the purpose of triage and screening (21). Belgium has taken legal steps in the direction of implementing novel triage instruments and allows some employees to be followed up at two-yearly intervals with a medical questionnaire in between, which serves to identify employees in need of closer follow-up (22). A new instrument has been developed especially to this end, which could be relevant for other countries as well (23).

In this study, we present the results of a quasi-randomized trial in four Belgian hospitals that compares care-as-usual (periodic health screenings by the occupational physician) with employees who complete an electronic health survey with selective follow-up (the intervention group). We compare the short-term effectiveness across four groups of outcomes: health, health-related productivity, healthcare use, and intermediate variables. The experiment coincided with the first two COVID-19 waves. As this affects both control and intervention groups equally, this allowed us to assess the effects of the intervention during a time that was, particularly in the hospital sector, characterized by heightened stress, work pressure, occupational health complaints, and population demand for health services (24).

## Methods

### Experimental design

The study protocol is published on ClinicalTrials.gov (Identifier NCT04684316). In a population of 53 Flemish hospitals, we recruited 4 large ones that were willing to participate in this study. In these hospitals, 3150 employees were eligible for periodic health screenings: personnel with safety functions, jobs with heightened vigilance, work that involves physical, biological or chemical agents or tasks that are an ergonomic or mental burden (22). Occupational groups that perform especially risky activities (frequent exposure to ionizing radiation, preparation of cytostatics, or exposure to carcinogens, mutagens, or reprotoxic substances) are excluded from the study population, as it is deemed that in these cases the electronic survey does not constitute adequate care that minimizes health risks.

We estimated that a minimum of 1700 employees (selected from four hospitals) had to be recruited (with an assumed dropout of 50%, effectively participating 850), equally divided over the two groups. This is grounded on power analyses in Stata 2014 MP, estimating sample size for a two-sample means test assuming equal standard deviations in intervention and control group, power 0.9 and significance level of 0.05, based on three data sources (25–27).

**Figure 1:**
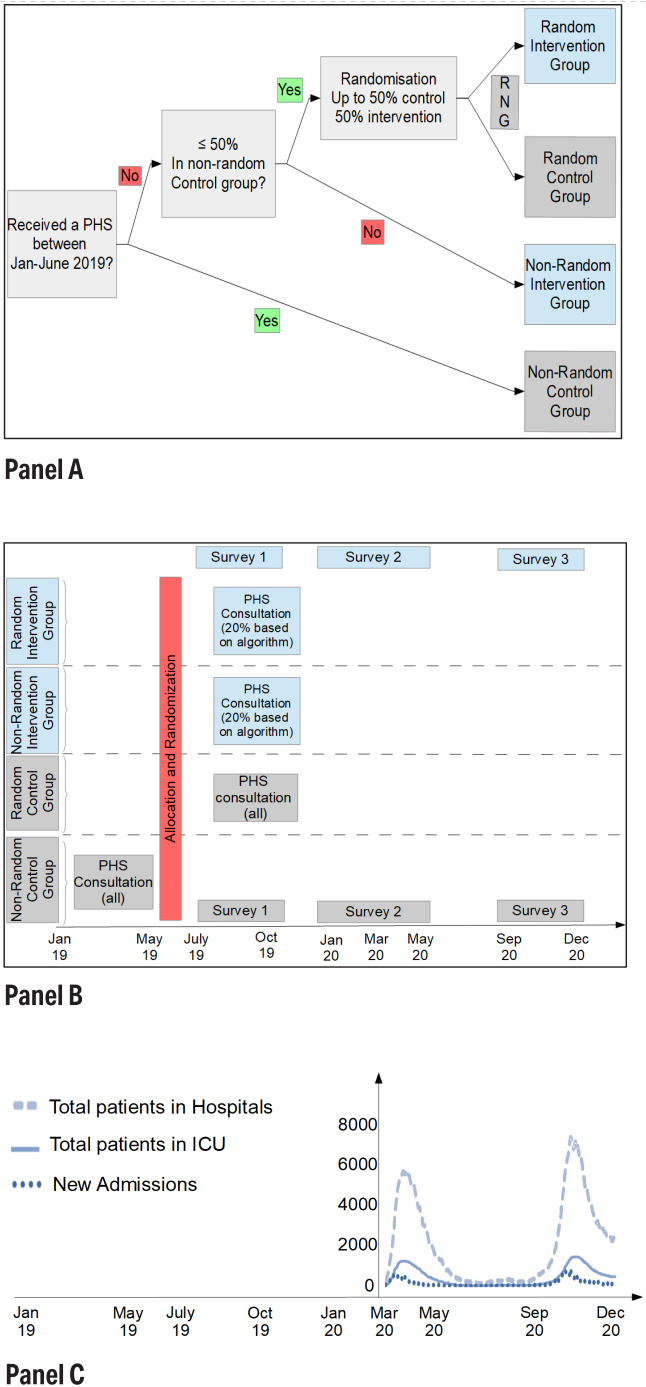
Study Design: panels A, B, and C. [RNG=random number generator; PHS=periodic health screening.] Panel A: Decision tree for subject allocation to groups. Panel B: Study design. Surveys for all participants between July – October 2019, January – May 2020, and September – December 2020. Non-random control group has a PHS consultation between January and June 2019. Other participants (random control group, and intervention group selected by algorithm) between July and December 2020. Panel C: Total number of patients, total patients in intensive care unit (ICU), and new Covid-19 admissions in Belgian hospitals during the study period. [source COVID-19 data: (28)].

Employees were allocated to one of four groups (figure 1, panel A): a random and non-random intervention group where employees received an electronic survey with selective occupational physician follow-up, and a random and non-random control group where all the occupational physician conducted periodic health screenings of employees. In May 2019, the majority of the participants were randomized (with a computerized random number generator) between control and intervention group until both groups were of equal size (1575 employees). However, some employees (who had gone the longest without a consultation) had already received a periodic health screening between January and June 2019, and were therefore non-randomly assigned to the control group. For one hospital, half of the participants were already allocated to the non-random control group in this way. Since randomization would have resulted in an intervention group with less than half of the participants, all remaining employees were (non-randomly) allocated to the intervention group.

The consultations (figure 1, panel B) occurred between January and June 2019 for the non-random control group, and between July and December 2019 for the other participants (random control group and employees of the intervention group that were referred by the algorithm). The quality of the randomization is evaluated by comparing the descriptive statistics of the control and intervention group and the samples against aggregate (population) data from the hospitals.

There are three measurement moments [each time with the same survey (23), and in both groups]: between June and October 2019 (before COVID-19), between February 2020 and May 2020 (seeing the start of the first COVID wave in Belgium), and between September 2020 and December 2020 (during the second peak in the hospitals). If participants in the intervention group did not complete the first survey, they were referred to the occupational physician for a consultation. The design is graphically represented in figure 1, panel B. The data for COVID-19 hospitalizations in Belgium and how this relates to the timing of the measurements is shown in figure 1, panel C. Both COVID-19 waves were notoriously large in Belgium, amongst the highest per capita in the world at the time (29).

In care-as-usual, all employees at risk were invited to attend a yearly screening, starting with a biometric examination, spirometry, vision test, and blood and urine test. The occupational physician then investigated the general health status and systems of the employee, which includes an anamnesis with questions about new health burdens or changes in occupational risks, follow-up questions on previous complaints, medical advice, referral to a healthcare provider, or booking another appointment with an occupational health specialist. In the intervention group, all employees completed an online health screening questionnaire. Dependent upon their answers, 20% of the employees [ie, the 20% of the employees who mostly needed contact with the occupational physician, as described elsewhere (23)] were referred to the occupational physician for a discussion of the results. For that 20%, the consultation then proceeded similar to care-as-usual. The other 80% did not receive further care.

In the control group, we expected that health screening would give rise to additional healthcare use in the short-term. The early detection and follow-up of health problems might then lead to a long-term amelioration in health, lower healthcare use and less absenteeism. In the intervention group, the short-term rise in healthcare use was expected to be smaller because there was less contact with an occupational physician, which could in turn lead to less long-term benefits.

However, these long-term effects often only occur after several years. Given the relatively brief timing of our data collection (19 months), we tried to circumvent this complication by also incorporating short-term outcomes. We expected the health literacy (25) and trust in physician to be higher in the control group compared to the intervention group, as these employees received more individual advice, and had an extra contact moment with the physician. We also expected turnover intention to be higher for the intervention group, as the occupational physician was less able to address job-specific issues. Finally, worry about health was expected to be higher in the intervention group as this is related to their health status.

Therefore, we compared the short-term effectiveness across four groups of outcomes over a follow-up period of 19 months: health, health-related productivity (absenteeism and presenteeism), healthcare, and intermediate variables (supplementary material, www.sjweh.fi/article/4011, supplement G). We focussed on three primary variables: general health [EuroQol 5-Dimension (EQ-5D] visual analog scale, 0–100), musculoskeletal problems (nordic musculoskeletal questionnaire, NMQ, 1–10), and general mental health (general health questionnaire, GHQ, 0–12). Secondary variables were absenteeism (days absent last four weeks), spontaneous consultations with the occupational physician (0–), health literacy score (HLS, 0–100), trust in physician (0–55), turnover intention, and a weighted score of worry intensity (how much do you worry about your health?), and frequency (how often do you worry about your health?). Supplementary tables S7–11 contain analyses for additional variables: stress (0–12), burnout (0–16), sleep problems (0–16), need for recovery at work (NFR, 0–11), referrals by the occupational physician (0–), work-related consultations with other providers (0–), job satisfaction (0–16), role conflicts (0–12), use of prescribed medication (no/yes), use of non-prescribed medication (no/yes), weighted presenteeism [multiplying days of reduced functioning by a functioning weight, as recommended in Bouwmans, Krol (30)].

### Statistical analysis

Generalised linear mixed effects models (GLMM) were used to assess the effect of the intervention upon the outcome measures. We discuss their nature, as well as the approach we took for our data, in depth in Supplement A. In our analyses, fixed effects were used on the level of the employee, and a random intercept and (time) slope effect was introduced to allow each employee to deviate from the (overall) fixed effect. The choice of the covariance structure (and random effects) for each outcome was based on the Akaike information criterion (AIC) (31).

If the quality of the sample indicated significant differences, we controlled for those covariates in the regression analyses, and made use of post-hoc Tukey-Kramer tests (also called honest significant difference tests) as a robustness test of our estimates (see Supplement A). Results were averaged over the levels of the covariates (hospital, gender, education), and a P-value adjustment was used by the Tukey method for comparing a family of 6 estimates (3× 2 groups).

In addition, non-inferiority and non-superiority tests of means were performed, with tests assuming a difference of delta (δ) affirms non-inferiority or non-superiority of the intervention mean versus the control mean. We thus verified whether the intervention was non-inferior (when higher outcomes are better) or non-superior (when higher outcomes are worse) in comparison to the control group (32). If the test was non-significant, non-inferiority or non-superiority cannot be concluded. Delta values were based on power calculations, clinically meaningful effects, and recommendations (32–34).

## Results

### Sample

In the four hospitals, 1077 unique employees participated in one or more of the three survey rounds and completely filled out the survey: 516 were allocated to the intervention group (441 random, 75 non-random), and 561 to the control group (81 random, 480 non-random). This means the average overall participation rate was 34% (1077 in 3150). Table 1 shows characteristics for wave 1 stratified by group. Supplementary table S1 shows the participation over the three survey rounds (N=776 for wave 1, N=418 for wave 2, N=588 for wave 3), and descriptive statistics by wave. Supplementary table S2 shows descriptive statistics for the 208 respondents (7%) that completed all three survey waves, and presents health status information.

**Table 1:** Baseline characteristics of the study population, stratified by group. Wave 1, January-October 2019, Ncontrol=396, Nintervention=380. [EQ-5D=EuroQol 5-Dimension; SD=standard devation]

Characteristics	Control Group (N=396)				Intervention Group (N=380)			
	
	N	%	Mean	SD	N	%	Mean	SD
Age			45.63	11.22			45.1	11.2
Gender								
Missing	1	0.3			1	0.3		
Male	78	19.7			65	17.1		
Female	317	80.1			314	82.6		
Education								
No degree	3	0.8			4	1.1		
Primary education	14	3.5			9	2.4		
Secondary education	85	21.5			63	16.6		
Higher education	294	74.2			299	78.7		
Other education	0	0.0			5	1.3		
EQ-5D visual analog scale			79.3	13.5			79.1	12.7
Musculoskeletal functioning			1.3	1.8			1.0	1.6
General mental health			2.0	2.9			2.2	2.9
Absenteeism last 4 weeks			0.6	2.7			0.2	1.3
Spontaneous consultations			0.3	0.9			0.1	0.6
Health literacy score			75.4	10.3			74.1	10.4
Trust in physician			38.4	6.0			37.2	6.3
Turnover intention			0.9	1.2			0.9	1.1
Worry weighted score			0.6	0.4			0.6	0.4

The 1077 unique employees gave rise to 1782 response records over all three measurement moments: 906 responses were from the control group, 876 from the intervention group. Of the 876 responses in the intervention group, 126 were referred by the algorithm to the consultation of an occupational physician (the top 20% of the scores), 684 were not referred (524 because they were in the bottom 80% of the scores, 160 because they missed the first measurement), and 66 were not referred but still opted for a face-to-face consultation. The 160 employees in the intervention group that missed the first measurement were invited for a face-to-face consultation with the physician, due to ethical reasons.

We assessed the quality of our sample in two ways. First, we evaluated the randomization process by verifying whether the control and intervention group differed substantially on background characteristics. Supplementary tables S3–5 show descriptive statistics by group for waves 1, 2, and 3, which demonstrate small differences for education in wave 1 (not wave 2 or 3). Second, we compared our sample with aggregated population data from hospitals B, C and D (there was no data available for hospital A), the results are presented in supplementary table S6. In light of these results, we controlled all regressions for age, gender, and education, but not occupation (since this is highly correlated to education), and made use of post-hoc tests.

### Generalised mixed models for final and intermediate outcomes

The estimates from the generalized mixed regressions are shown in tables 2–3, full models are available in supplementary tables S7–11, and show the average fixed effects for group and time, and the interaction effects. Note how the absence of COVID-19 makes the first measurement (pre-COVID) fundamentally different from the second and third measurements.

**Table 2:** Estimation results from mixed-effects models for primary outcomes. [ CI=confidence interval; EQ-5D=EuroQol 5-Dimension; LME=linear mixed effects]

Dependent variable	EQ-5D vas	Musculoskeletal functioning (log)	General mental health (log)
		
Estimates	LME ^a^ (95% CI)	LME ^a^ (95% CI)	LME ^a^ (95% CI)
Intervention group (ref.=control)	-0.26 (-2.01–1.49)	-0.06 (-0.15–0.02)	0.07 (-0.04–0.19)
Time 2 (ref.=Time 1) ^b^	-1.72 ^c^ (-3.50–0.05)	-0.12 ^d^(-0.20– -0.04)	0.08 (-0.04–0.19)
Time 3 ^b^	-1.11 (-2.80–0.57)	0.45 ^d^ (0.36–0.54)	0.17 ^d^ (0.05–0.28)
Intervention: time 2	-0.46 (-2.94–2.01)	0.13 ^e^ (0.02–0.24)	-0.10 (-0.26–0.06)
Intervention: time 3	0.00 (-2.41–2.41)	0.01(-0.12– 0.14)	-0.15 ^a^ (-0.32–0.01)
Observations	1733	1736	1736
Range	(0–100)	(1–10)	(1–13)

a Estimates are controlled for age, gender, educational attainment, and hospital, with a random intercept and slope by employee.

b Time 1 = first measurement between June and October 2019, Time 2 = second measurement between February 2020 and May 2020, Time 3 = third measurement between September 2020 and December 2020.

c P<0.1.

d P<0.01.

e P<0.05.

**Table 3:** Estimation results from mixed-effects models for secondary outcomes. [CI=confidence interval; IRR=incidence rate ratios; LME=linear mixed effects; OR=odds ratio]

Dependent variable	Absenteeism last 4 weeks	Spontaneous consultations	Health literacy	Trust in physician	Turnover intention	Worry weighted score
					
Estimates	IRR ^a^ (95% CI)	IRR ^b^ (95% CI)	LME ^c^ (95% CI)	LME ^c^ (95% CI)	LME ^c^ (95% CI)	LME ^c^ (95% CI)
Intervention group (ref.=control)	0.30 ^e^ (0.14–0.63)	0.35 ^e^(0.23–0.55)	-0.97 (-2.43–0.49)	-0.94 ^f^ (-1.78– -0.10)	-0.02 (-0.17–0.13)	-0.02 (-0.07–0.03)
Time 2 (ref.=Time1) ^d^	1.36 (0.74–2.51)	0.54 ^e^ (0.34–0.86)	-1.31 ^g^(-2.62– -0.00)	-0.32 (-0.99–0.36)	0.19 ^e^ (0.05–0.32)	-0.03 (-0.08–0.01)
Time 3 ^d^	1.38 (0.74–2.59)	0.59 ^e^ (0.40–0.87)	1.22 ^g^ (-0.19–2.63)	-0.04 (-0.79–0.72)	-0.15 ^f^ (-0.28– -0.02	0.03 (-0.01–0.08)
Intervention: time 2	4.11 ^e^ (1.69–10.01)	2.51 ^f^ (1.20–5.27)	1.55 ^g^ (-0.27–3.38)	0.32 (-0.61–1.25)	-0.18 ^g^ (-0.37–0.00	0.07 ^f^ (0.01–0.13)
Intervention: time 3	2.96 ^f^ (1.11–7.89)	2.47 ^e^ (1.29–4.75)	-0.36 (-2.38–1.65)	0.76 (-0.32–1.85)	0.15 (-0.04–0.34)	-0.00 (-0.07–0.06)
Observations	1684	1736	1688	1642	1723	1735
Range	(0–28)	(0–5)	(0–100)	(0–55)	(0–4)	(0–1.55)

a Estimates are controlled for age, gender, educational attainment, and hospital. The underlying Generalised Poisson model contains a zero-inflated intercept and dispersion parameter, but no random effects.

b Estimates are controlled for age, gender, educational attainment, and hospital. The underlying Negative Binomial model contains a dispersion parameter but no random effects.

c Estimates are controlled for age, gender, educational attainment, and hospital, with a random intercept and slope by employee.

d Time 1 = first measurement between June and October 2019, Time 2 = second measurement between February 2020 and May 2020, Time 3 = third measurement between September 2020 and December 2020.

g P<0.1.

f P<0.05.

e P<0.01.

The results indicate no significant overall differences between intervention and control group for health status (EQ-5D vas), musculoskeletal problems, general mental health, health literacy, turnover intention, and worrying about health. The intervention group had, on average, lower scores for trust in the physician (-0.94 on a scale of 55. The intervention group was also, on average, fewer days absent from work [incident rate ratio (IRR) 0.30], and had fewer spontaneous consultations (IRR=0.35). However, these were mainly the result of differences in the first measurement.

The interactions at time 2 indicate an increase in the difference of intervention and control group for musculoskeletal problems, health literacy, the weighted worry-score, and (because turnover intention in the control group increased) a decrease in the difference of turnover intention, compared to the difference of intervention and control group in time 1.

At the point of final measurement, general mental health is reduced for the intervention group. The between group differences of absenteeism and spontaneous consultations decreased (the intervention group rose in comparison to the control group in time 1, making the difference smaller). The interactions thus indicate a short-term difference between groups at time 1 that diminished over time. We judged the absence of effects in time 2 and 3 as more important than the presence of group differences in time 1, as we hypothesized that the occupational burdens to which participants were exposed were higher during COVID-19 (time 2 and 3).

Supplementary tables S12–13 summarize the post-hoc estimations. For the superiority post-hoc differences, only absenteeism, referrals, and spontaneous consultations are significantly different between groups (first three columns of supplementary table S12). This is represented graphically in supplementary figure S1. In the non-inferiority tests, the majority of the estimates indicate the intervention is at least as good as care-as-usual (EQ-5D, NMQ, stress, GHQ, NFR, HLS, trust, job satisfaction, absenteeism, healthcare use, and presenteeism). If the null hypothesis cannot be rejected, this does not indicate that the reverse (inferiority of the intervention group) is true.

## Discussion

Non-inferiority tests indicate the intervention group performs at least as well as the control group on the majority of the outcomes. The regressions and (superiority) post-hoc analyses indicate that there were some short-term effects of the intervention: trust in the physician, absenteeism and healthcare use (referrals, spontaneous consultations, and work-related consultations) were lower in the intervention group. However, these differences predominantly occurred in wave 1 and were no longer present in the third measurement moment when participants had been exposed to exceptional circumstances.

Because the second and third measurement largely coincided with peaks in COVID-19 hospitalizations, the COVID pandemic possibly increased the healthcare needs of hospital personnel during this period (24). We believe this was an unforeseen advantage of our study: whereas in normal situations it can take a long time before employees develop complaints, the external health shock accelerates this process, as employees were temporarily put under extreme strain. However, while our data and other publications (24) suggests this is the case, a causal interpretation is subject to discussion: personnel might have been more committed to their patients’ care than usual; as operations were postponed, workload might have decreased, and not all participants were medical personnel.

In a way, the absence of large differences between the control and intervention group could have been expected for some of the outcomes (eg, overall health): it often takes a long time before the beneficial effects of screening become apparent, and our study duration (19 months) might have been too short to pick this up. Although the COVID-19 pandemic had a clear impact on the data – for instance visible in the increased stress, burnout risk, and musculoskeletal functioning problems over time – it did not increase the differences between the face-to-face screenings of the control group compared to the targeted screenings. While outcomes changed over time, differences between groups remained largely absent.

The differences we did find (absenteeism, trust, and healthcare use) were mainly driven by differences in the first measurement. Their direction seems plausible: after the control group had a face-to-face screening, it seems natural that their trust in their physician would increase. Likewise, if health or functioning problems are discussed in the screening, this might lead to additional consultations (hence increased healthcare use) or increased absenteeism (eg, to consult specialists or by following the physician’s advice to stay home). The rise in healthcare use is consistent with other literature, where a higher healthcare use is one of the short-term consequences of face-to-face screening, but the rise in absenteeism is not (27). The fact that these effects are no longer present in the second and third wave might be explained by several factors. COVID-19 might have urged personnel (from control and intervention groups alike) to refrain from taking absence leave because of the increased pressure on the hospitals or from visiting physicians because of contact restrictions. In addition, it is also possible that the effects of face-to-face contact were only temporary: an immediate increase in healthcare use, absenteeism, and trust that faded away by the time of the second and third measurement. Third, the non-perfect randomization might have played a role, although the robustness checks of the quality of our sample (both vis-à-vis the population and based on background differences between groups) did not indicate large problems in this respect.

Some limitations and potential biases should also be pointed out. Although we verified the quality of our sample, the randomization process was only partial and participation could have been affected by selection bias. Similarly, our results are dependent on the four selected hospitals, although care was taken to select large hospitals from different regions. The results are also limited to the short timeframe of the study (19 months), limited participation (13–25% across waves, 7% completed all surveys) and to the use of self-assessed outcomes. Because self-assessed outcomes were used, in contrast to diagnostic information from occupational physicians, this study focused on the effectiveness of the intervention, not on its ability to detect health problems (eg, burnouts). The design and performance of the survey and algorithm was however taken up in a previous article (23). Finally, it is possible that the intervention (leaving out a face-to-face screening with the physician) had little effect because the employee and physician already had a long-standing bond, built up from past screenings and workplace visits. As shown in supplementary tables S1–2, about half the sample knew the occupational physician for five years or longer. A robustness analysis where this relation was added as a fixed covariate effect did not show substantial differences.

While COVID-19 might have increased the burden on personnel and gives an indication of long-term effects, the intervention’s short-term effects might differ in a non-COVID-19 period, and the true long-term effects should be measured in further research. It is still possible that, as in other prevention studies, the benefits of face-to-face screening only become apparent in the long run (35) [eg, diseases with a long latency period such as silicosis (36)]. Nevertheless, the short-term impact of screening is far from irrelevant: turnover is traditionally high in the hospital sector (causing short employment periods), intermediate outcomes give an indication of long-term effects, and short-term changes can strongly affect cost-effectiveness (eg, a higher healthcare use in the short term without long-term improvements can point to overuse of care).

For policy-makers and practitioners, we want to emphasize that we did not compare physician screening with no screening but rather more targeted screening. This means we cannot make claims on the effectiveness of physician screening, but can only compare it to the intervention: screening after an additional risk selection. In addition, we wish to highlight that occupational health services and periodic health screenings can have other purposes – they can be used for primary, secondary, and tertiary prevention. Our conclusions are limited to the measured outcomes and timeframe of 19 months.

### Concluding remarks

For those populations where physical consultations are not strictly necessary, a lower frequency of face-to-face health screening might prove at least as effective if combined with a more targeted approach to ensure those who really need it are still offered adequate care.

## Supplementary material

Supplementary material
