# Supplementary material for: Short-term effectiveness of face-to-face periodic occupational health screening versus electronic screening with targeted follow-up: results from a quasi-randomized controlled trial in four Belgian hospitals
Source: Scand J Work Environ Health. 2022 Mar 31;48(3):220–8. doi: 10.5271/sjweh.4011 (PMC9523468; doi:10.5271/sjweh.4011)
Supplement: Supplementary material [file SJWEH-48-220-S001.pdf]

Short-term effectiveness of face-to-face periodic occupational health screening versus electronic screening with targeted follow-up: results from a quasi-randomized controlled trial in four Belgian hospitals[1]

by Jonas Stefaan Steel, PhD,[2] Lode Godderis, MD, PhD, Jeroen Luyten, PhD

1. *SUPPLEMENTARY FILES*
2. *Correspondence to: Jonas Steel, Leuven Institute for Healthcare Policy, KU Leuven, Kapucijnenvoer 35, 3000 Leuven, Belgium. [E-mail: jonas.steel@kuleuven.be]*

Supplement A: Generalised Mixed Effects Models

Generalised Linear Mixed Effects models (GLMM) were used to assess the effect of the intervention upon the outcome measures. We base the following paragraphs on (31, 37, 38). In its non-generalised form, the linear mixed effect model (LMM) is an extension of simple linear models by allowing for both fixed and random effects. The fixed effects are introduced on the highest level of the data, are the same for all subjects, and capture between group differences. The random effects are subject-specific and capture deviations from the fixed effects, and thus capture part of the within group variability. Analogous to subjects randomly drawn from a population of subjects, the subject-specific random effects can be considered as randomly sampled from a population of regression coefficients, often with an assumed underlying distribution (e.g. a multivariate normal distribution). In its hierarchical form, the linear mixed effect model (LMM) can be represented as follows (37):

$$\begin{cases} \boldsymbol{Y_i} = \boldsymbol{X_i\beta} + \boldsymbol{Z_i b_i} + \boldsymbol{\varepsilon_i} \\ \boldsymbol{b_i} \sim \mathcal{N}(\boldsymbol{0}, \boldsymbol{D}) \\ \boldsymbol{\varepsilon_i} \sim \mathcal{N}(\boldsymbol{0}, \boldsymbol{\Sigma_i}) \\ \boldsymbol{b_i}, \dots \boldsymbol{b_n}, \boldsymbol{\varepsilon_i}, \dots, \boldsymbol{\varepsilon_n} \quad \text{independent} \end{cases} \tag{1}$$

with $\mathbf{Y_i}$ the response vector of repeated measurements for subject i ($1 \leq i \leq N$, N number of subjects), $\mathbf{X_i}$ and $\mathbf{Z_i}$ matrices of covariates, $\boldsymbol{\beta}$ a vector with the fixed effects, $\mathbf{b_i}$ a vector of random effects, and $\boldsymbol{\varepsilon_i}$ the residuals. Note how $\boldsymbol{\beta}$ does not have a subscript i (the parameters are the same for all subjects), but $\mathbf{b_i}$ does (parameters vary over subjects). $\mathbf{D}$ and $\boldsymbol{\Sigma_i}$ are covariance matrices; they imply a flexible and specific covariance structure that allows for highly unbalanced data. Equation (1) can also be stated as follows (31):

$$\begin{cases} \mathbf{Y_i}|\mathbf{b_i} \sim \mathcal{N}(\mathbf{X_i}\boldsymbol{\beta} + \mathbf{Z_i b_i}, \boldsymbol{\Sigma_i}) \\ \mathbf{b_i} \sim \mathcal{N}(\mathbf{0}, \mathbf{D}) \\ \mathbf{Y_{ij}}|\mathbf{b_i} \quad \text{independent} \end{cases} \tag{2}$$

In a non-Bayesian framework, estimating (2) is based on the marginal distribution for $\mathbf{Y_i}$ (integrating out the random effects) through maximum likelihood, which renders consistent, asymptotic normal, and efficient estimates. The model-fitting procedures are iterative and Newton-Raphson-based, and are described in the R package 'nlme' documentation (39, 40).

Analogous to univariate standard and generalised linear models, the linear mixed model (LMM) in Equation (1) and (2) is a specific case of generalised linear mixed models (GLMM). In GLMM, $\mathbf{Y_{ij}}$ have densities $f_i(y_{ij}|\theta_{ij},\phi)$ of the exponential family (e.g. normal, Poisson, generalised Poisson, negative binomial, or binomial distributed dependent variables) with θ a 'natural' parameter and ϕ a 'scale' parameter. GLMM models can then be represented as follows (31):

$$\begin{cases} \mathbf{Y_i}|\mathbf{b_i} \sim \text{Exp}_i(\boldsymbol{\theta},\boldsymbol{\phi}) \\ \mathbf{b_i} \sim \mathcal{N}(\mathbf{0},\mathbf{D}) \\ \mathbf{Y_{ij}}|\mathbf{b_i} \quad \text{independent} \end{cases} \tag{3}$$

To estimate (3), the mean μ is then modelled for a link function $\eta(\cdot)$: $(\mu_{ij}) = \eta[E(Y_{ij}|\mathbf{b_i})] = \mathbf{x'_{ij}}\boldsymbol{\beta} + \mathbf{z'_{ij}}\mathbf{b_i}$. If the natural link function is used $\eta(\cdot) = \psi'(\cdot)$, this renders $\boldsymbol{\theta_{ij}} = \mathbf{x'_{ij}}\boldsymbol{\beta} + \mathbf{z'_{ij}}\mathbf{b_i}$, which satisfies a linear regression model.

If $\mathbf{Y_i}|\mathbf{b_i}$ is normally distributed with mean $\mathbf{X_i}\boldsymbol{\beta} + \mathbf{Z_i}\mathbf{b_i}$, and covariance $\boldsymbol{\Sigma_i}$, Equation (3) collapses to (2). The density $f_i(y_{ij})$ is then the normal distribution, with $\theta = \mu$, $\phi = \sigma^2$, and variance function $v(\mu) = 1$. If $f_i(y_{ij})$ is the Poisson density with $\theta = ln\lambda$, $\phi = 1$, variance function $v(\lambda) = \lambda$, and log link function, a Poisson mixed model can be estimated, or a negative binomial model when an extra dispersion parameter is added. If $f_i(y_{ij})$ is a Bernoulli density, and using a probit or logit link function, a probit or logit normal model can be obtained.

On the first level (L1), the β has subscripts (j) to indicate they belong to one particular employee. On the second level, each β for a particular employee (β_{pj}) can be represented as a combination of a mean estimate for that parameter (γ_{p0}), and a random effect for that measurement moment (u_{pj}). Only the intercept (β_{0j}) and time slope (β_{2j}) can vary across employees, the other β_{pj} are fixed.

$$L1: Y_{ij} = \beta_{0j} + \beta_{1j}Group_{ij} + \beta_{2j}Time_{ij} + \beta_{3j}Group * Time_{ij} + \beta_{pj}Covariates_{ij} + e_{ij} \tag{4.1}$$

$$L2: \begin{cases} \beta_{0j} = \gamma_{00} + u_{0j} \\ \beta_{1j} = \gamma_{10} \\ \beta_{2j} = \gamma_{20} + u_{2j} \\ \beta_{3j} = \gamma_{30} \\ \beta_{pj} = \gamma_{p0} \end{cases} \tag{4.2}$$

with $\beta_{pj}Covariates_{ij}$ containing age, gender, hospital, and education. In each model, fixed effects were thus included for group (intervention or control) and time (1,2,3), covariate fixed

effects for age, gender, educational attainment, and hospital, and random intercept effects for each individual employee.

Approach in our analyses

In our data, some of the measured outcomes are typically left- or right-skewed. We therefore used logarithmic transformations for musculoskeletal complaints (NMQ), general mental health (GHQ), and need for recovery (NFR). We also make use of continuous ordinal regression (41) to analyse the visual analog scale for general health status (EQ-5D), as several authors have argued that it should be analysed as an ordinal instead of a continuous variable ((42-45) as cited in (41)). As this method is not standardly used, we also include the linear mixed model of the EQ-5D visual analog scale.

It is moreover often likely that count variables diverge from the normality assumption of the residuals because of frequent zero-values (e.g. zero days absent, zero consultations, etc.) and dispersed non-zero values (long tails). We therefore estimated these outcomes with binomial and Poisson-type (e.g. generalised Poisson or negative binomial) mixed models. The fit of these models is tested by several tests for simulated residuals (46): a Kolmogorov–Smirnov test (KS) to test distributional fit, a test for the presence of zero-inflation, and a dispersion test to verify whether the observed variance is higher or lower than the theoretical variance of the model. A generalised Poisson or negative binomial model is adopted when it is necessary to specify a separate dispersion parameter to help alleviate over- or under-dispersion. We also test whether a large number of outliers occur, and quantiles are compared to their theoretical value. Finally, the presence of autocorrelation is tested with a Durbin-Watson test on uniformly scaled residuals , and the covariance structure is adjusted (if autocorrelation is present).

Post-hoc tests

After the analyses, we make use of post-hoc Tukey-Kramer tests (also called honest significant difference tests) as a robustness test of our estimates.

$$Tukey = \frac{M_i - M_j}{\sqrt{\frac{MS_w}{n_h}}} \quad (5)$$

where $M_i - M_j$ is the difference between the estimated means, MS_w is the Mean Square Within, and n is the number of employees in the group. The means are obtained by making predictions on the estimated mixed models (e.g. the mean stress score for the control and intervention group in time 1, 2, and 3) with their standard deviation. The pairwise differences of these six (3 times * 2 groups) means are then compared. Results are averaged over the levels of the covariates (hospital, gender, education), and a p-value adjustment is used by the Tukey method for comparing a family of 6 estimates. Because the sample sizes are unequal (unbalanced panel data), the Tukey-Kramer adjustment is used to estimate separate standard deviations for each pairwise comparison. A similar approach is used for the non-inferiority tests.

References supplement A

1. Verbeke G, Molenberghs G. Linear mixed models for longitudinal data. New York: Springer; 2000 2000.

2. UCLA Insitute for Digital Research Education,. Introduction to Linear Mixed Models.

3. Molenberghs G, Verbeke G. Models for Discrete Longitudinal Data. New York, NY: Springer New York; 2006.

4. Pinheiro J, Bates D, DebRoy S, Sarkar D, Team RC. nlme: Linear and Nonlinear Mixed Effects Models 2021.

5. Lindstrom MJ, Bates DM. Newton—Raphson and EM algorithms for linear mixed-effects models for repeated-measures data. Journal of the American Statistical Association. 1988;83(404):1014-22.

6. Manuguerra M, Heller GZ, Ma J. Continuous Ordinal Regression for Analysis of Visual Analogue Scales: The R Package ordinalCont. Journal of Statistical Software. 2020;96(1):1-25.

7. Philip BK. Parametric statistics for evaluation of the visual analog scale. Anesthesia & Analgesia. 1990;71(6):710.

8. Wewers ME, Lowe NK. A critical review of visual analogue scales in the measurement of clinical phenomena. Research in nursing & health. 1990;13(4):227-36.

9. Svensson E. Comparison of the quality of assessments using continuous and discrete ordinal rating scales. Biometrical Journal: Journal of Mathematical Methods in Biosciences. 2000;42(4):417-34.

10. Kersten P, Küçükdeveci AA, Tennant A. The use of the Visual Analogue Scale (VAS) in rehabilitation outcomes. Journal of rehabilitation medicine. 2012;44(7):609-10.

11. Hartig F, Hartig MF. Package ‘DHARMa’ 2017.

Supplement B: descriptive statistics by wave

Supplemental Table SS1. Summary Statistics by wave: mean (proportion)

Time	Wave 1 (N=776)	Wave 2 (N=418)	Wave 3 (N=588)	Total (N=1782)
Gender				
Missing	2	16	27	45
Male	143 (18.5%)	79 (19.7%)	107 (19.1%)	329 (18.9%)
Female	631 (81.5%)	323 (80.3%)	454 (80.9%)	1408 (81.1%)
Other	0 (0.0%)	0 (0.0%)	0 (0.0%)	0 (0.0%)
Age				
Missing	0	15	26	41
Mean (SD)	45.387 (11.197)	46.757 (11.203)	46.797 (10.686)	46.159 (11.052)
Range	17.000 - 66.000	18.000 - 65.000	18.000 - 65.000	17.000 - 66.000
Income				
Missing	5	1	34	40
Income less than 1500 monthly	123 (16.0%)	47 (11.3%)	80 (14.4%)	250 (14.4%)
Income 1500,00 - 1999,99 monthly	240 (31.1%)	127 (30.5%)	167 (30.1%)	534 (30.7%)
Income 2000,00 - 2499,99 monthly	241 (31.3%)	127 (30.5%)	186 (33.6%)	554 (31.8%)
Income 2500,00 - 2999,99 monthly	76 (9.9%)	48 (11.5%)	62 (11.2%)	186 (10.7%)
Income 3000,00 - 4999,99 monthly	26 (3.4%)	12 (2.9%)	22 (4.0%)	60 (3.4%)
Income > 5000,00 monthly	5 (0.6%)	3 (0.7%)	2 (0.4%)	10 (0.6%)
Income not stated	60 (7.8%)	53 (12.7%)	35 (6.3%)	148 (8.5%)
Nationality				
Missing	0	0	17	17
Belgian	763 (98.3%)	399 (99.0%)	557 (99.1%)	1719 (98.7%)
Migrant inside EU	9 (1.2%)	3 (0.7%)	5 (0.9%)	17 (1.0%)
Migrant outside EU	4 (0.5%)	1 (0.2%)	0 (0.0%)	5 (0.3%)
Education				
Missing	0	15	27	42
No degree	7 (0.9%)	0 (0.0%)	1 (0.2%)	8 (0.5%)

Time	Wave 1 (N=776)	Wave 2 (N=418)	Wave 3 (N=588)	Total (N=1782)
Primary education	23 (3.0%)	9 (2.2%)	17 (3.0%)	49 (2.8%)
Secondary education	148 (19.1%)	75 (18.6%)	98 (17.5%)	321 (18.4%)
Higher education	593 (76.4%)	316 (78.4%)	441 (78.6%)	1350 (77.6%)
Other education	5 (0.6%)	3 (0.7%)	4 (0.7%)	12 (0.7%)
Household				
Missing	0	15	26	41
Single	91 (11.7%)	42 (10.4%)	55 (9.8%)	188 (10.8%)
Single with child(ren)	63 (8.1%)	26 (6.5%)	43 (7.7%)	132 (7.6%)
Couple with child(ren)	486 (62.6%)	265 (65.8%)	373 (66.4%)	1124 (64.6%)
Couple without child(ren)	130 (16.8%)	66 (16.4%)	88 (15.7%)	284 (16.3%)
Other	6 (0.8%)	4 (1.0%)	3 (0.5%)	13 (0.7%)
Occupation				
Missing	27	4	4	35
Medical Personnel	394 (52.6%)	212 (51.2%)	319 (54.6%)	925 (52.9%)
Paramedics	20 (2.7%)	20 (4.8%)	24 (4.1%)	64 (3.7%)
Technicians	56 (7.5%)	35 (8.5%)	40 (6.8%)	131 (7.5%)
Administrative Personnel	120 (16.0%)	75 (18.1%)	93 (15.9%)	288 (16.5%)
Management	34 (4.5%)	22 (5.3%)	27 (4.6%)	83 (4.8%)
Cleaning Staff	34 (4.5%)	12 (2.9%)	24 (4.1%)	70 (4.0%)
Other	91 (12.1%)	38 (9.2%)	57 (9.8%)	186 (10.6%)
Hospital				
Hospital A	197 (25.4%)	101 (24.2%)	175 (29.8%)	473 (26.5%)
Hospital B	266 (34.3%)	113 (27.0%)	147 (25.0%)	526 (29.5%)
Hospital C	214 (27.6%)	125 (29.9%)	193 (32.8%)	532 (29.9%)
Hospital D	99 (12.8%)	79 (18.9%)	73 (12.4%)	251 (14.1%)
Group				
Non-random control	340 (43.8%)	187 (44.7%)	266 (45.2%)	793 (44.5%)
Random control	56 (7.2%)	15 (3.6%)	42 (7.1%)	113 (6.3%)
Non-random intervention	53 (6.8%)	44 (10.5%)	35 (6.0%)	132 (7.4%)

Time	Wave 1 (N=776)	Wave 2 (N=418)	Wave 3 (N=588)	Total (N=1782)
Random intervention	327 (42.1%)	172 (41.1%)	245 (41.7%)	744 (41.8%)
Language				
Missing	434	169	0	603
Dutch	330 (96.5%)	240 (96.4%)	569 (96.8%)	1139 (96.6%)
French	3 (0.9%)	3 (1.2%)	3 (0.5%)	9 (0.8%)
Other	9 (2.6%)	6 (2.4%)	16 (2.7%)	31 (2.6%)
Absenteeism				
Missing	4	8	11	23
Mean (SD)	4.807 (31.922)	1.229 (4.720)	1.007 (5.034)	2.727 (21.535)
Range	0.000 - 526.000	0.000 - 57.000	0.000 - 90.000	0.000 - 526.000
Prescribed.medication.use..last.2.weeks.				
Missing	0	1	1	2
No	379 (48.8%)	199 (47.7%)	298 (50.8%)	876 (49.2%)
Yes	397 (51.2%)	218 (52.3%)	289 (49.2%)	904 (50.8%)
Non.prescribed.medication.use..last.2.weeks.				
Missing	1	1	0	2
No	525 (67.7%)	256 (61.4%)	357 (60.7%)	1138 (63.9%)
Yes	250 (32.3%)	161 (38.6%)	231 (39.3%)	642 (36.1%)
Year.of.last.PHS				
Missing	22	16	46	84
Never	12 (1.6%)	5 (1.2%)	7 (1.3%)	24 (1.4%)
2009 or earlier	1 (0.1%)	3 (0.7%)	0 (0.0%)	4 (0.2%)
2010	0 (0.0%)	1 (0.2%)	0 (0.0%)	1 (0.1%)
2011	0 (0.0%)	0 (0.0%)	0 (0.0%)	0 (0.0%)
2012	2 (0.3%)	0 (0.0%)	0 (0.0%)	2 (0.1%)
2013	0 (0.0%)	0 (0.0%)	0 (0.0%)	0 (0.0%)
2014	4 (0.5%)	0 (0.0%)	0 (0.0%)	4 (0.2%)
2015	6 (0.8%)	2 (0.5%)	1 (0.2%)	9 (0.5%)
2016	13 (1.7%)	5 (1.2%)	5 (0.9%)	23 (1.4%)

Time	Wave 1 (N=776)	Wave 2 (N=418)	Wave 3 (N=588)	Total (N=1782)
2017	88 (11.7%)	17 (4.2%)	15 (2.8%)	120 (7.1%)
2018	314 (41.6%)	123 (30.6%)	86 (15.9%)	523 (30.8%)
2019	314 (41.6%)	246 (61.2%)	252 (46.5%)	812 (47.8%)
2020	0 (0.0%)	0 (0.0%)	176 (32.5%)	176 (10.4%)
Time.since.last.PMO				
Missing	34	21	53	108
0	314 (42.3%)	0 (0.0%)	176 (32.9%)	490 (29.3%)
1	314 (42.3%)	246 (62.0%)	252 (47.1%)	812 (48.5%)
2	88 (11.9%)	123 (31.0%)	86 (16.1%)	297 (17.7%)
3	13 (1.8%)	17 (4.3%)	15 (2.8%)	45 (2.7%)
4	6 (0.8%)	5 (1.3%)	5 (0.9%)	16 (1.0%)
5	4 (0.5%)	2 (0.5%)	1 (0.2%)	7 (0.4%)
7	2 (0.3%)	0 (0.0%)	0 (0.0%)	2 (0.1%)
10	1 (0.1%)	1 (0.3%)	0 (0.0%)	2 (0.1%)
11	0 (0.0%)	3 (0.8%)	0 (0.0%)	3 (0.2%)
Turnover.intention				
Missing	8	2	4	14
Mean (SD)	0.924 (1.155)	0.945 (1.230)	0.841 (1.154)	0.902 (1.173)
Range	0.000 - 4.000	0.000 - 4.000	0.000 - 4.000	0.000 - 4.000
Relation.with.OP				
Missing	2	0	3	5
I have not yet met the occupational physician	43 (5.6%)	14 (3.3%)	23 (3.9%)	80 (4.5%)
Less than 1 year	104 (13.4%)	34 (8.1%)	34 (5.8%)	172 (9.7%)
1 to 2 years	165 (21.3%)	81 (19.4%)	100 (17.1%)	346 (19.5%)
3 to 4 years	135 (17.4%)	84 (20.1%)	111 (19.0%)	330 (18.6%)
5 years or more	327 (42.2%)	205 (49.0%)	317 (54.2%)	849 (47.8%)
Work.related.HC				
Missing	0	0	2	2
Mean (SD)	1.384 (2.115)	1.299 (2.098)	1.314 (2.074)	1.341 (2.097)

Time	Wave 1 (N=776)	Wave 2 (N=418)	Wave 3 (N=588)	Total (N=1782)
Range	0.000 - 6.000	0.000 - 6.000	0.000 - 6.000	0.000 - 6.000
Referrals.by.OP.to.other.HC				
Missing	1	0	2	3
Mean (SD)	0.263 (1.003)	0.165 (0.801)	0.213 (0.966)	0.224 (0.947)
Range	0.000 - 6.000	0.000 - 6.000	0.000 - 6.000	0.000 - 6.000
Spontaneous.consultations.with.OP				
Mean (SD)	0.232 (0.750)	0.144 (0.557)	0.190 (0.632)	0.198 (0.671)
Range	0.000 - 6.000	0.000 - 6.000	0.000 - 6.000	0.000 - 6.000
Health.Literacy.Score				
Missing	25	8	19	52
Mean (SD)	74.779 (10.407)	74.825 (10.133)	76.030 (11.336)	75.201 (10.669)
Range	37.500 - 100.000	25.000 - 100.000	25.000 - 100.000	25.000 - 100.000
Trust.in.Physician.Score				
Missing	51	16	29	96
Mean (SD)	37.851 (6.170)	37.418 (6.341)	38.338 (6.491)	37.909 (6.324)
Range	14.000 - 55.000	11.000 - 55.000	11.000 - 55.000	11.000 - 55.000
Stress.score				
Mean (SD)	4.013 (2.528)	4.201 (2.484)	4.366 (2.441)	4.173 (2.493)
Range	0.000 - 12.000	0.000 - 12.000	0.000 - 12.000	0.000 - 12.000
General.Mental.Health				
Mean (SD)	2.067 (2.900)	2.153 (3.030)	2.468 (3.222)	2.219 (3.043)
Range	0.000 - 12.000	0.000 - 12.000	0.000 - 12.000	0.000 - 12.000
Need.for.Recovery				
Mean (SD)	3.772 (2.787)	3.632 (2.659)	3.952 (2.873)	3.799 (2.788)
Range	0.000 - 11.000	0.000 - 10.000	0.000 - 11.000	0.000 - 11.000
Sleep.score				
Mean (SD)	5.174 (3.673)	4.998 (3.649)	5.427 (3.731)	5.216 (3.688)
Range	0.000 - 16.000	0.000 - 16.000	0.000 - 16.000	0.000 - 16.000

Time	Wave 1 (N=776)	Wave 2 (N=418)	Wave 3 (N=588)	Total (N=1782)
Role.conflicts				
Mean (SD)	3.620 (2.548)	3.651 (2.575)	3.612 (2.586)	3.625 (2.566)
Range	0.000 - 12.000	0.000 - 12.000	0.000 - 12.000	0.000 - 12.000
Job.satisfaction				
Missing	2	0	0	2
Mean (SD)	15.235 (2.377)	15.187 (2.483)	15.224 (2.317)	15.220 (2.382)
Range	6.000 - 20.000	7.000 - 20.000	7.000 - 20.000	6.000 - 20.000

Supplemental Table SS2. Summary Statistics by wave: complete cases: mean (proportion)

Time	Wave 1 (N=208)	Wave 2 (N=208)	Wave 3 (N=208)	Total (N=624)
Gender				
- Missing	1	1	1	3
- Male	49 (23.7%)	49 (23.7%)	49 (23.7%)	147 (23.7%)
- Female	158 (76.3%)	158 (76.3%)	158 (76.3%)	474 (76.3%)
- Other	0 (0.0%)	0 (0.0%)	0 (0.0%)	0 (0.0%)
Age				
- Mean (SD)	47.553 (10.454)	48.553 (10.454)	48.553 (10.454)	48.220 (10.448)
- Range	17.000 - 63.000	18.000 - 64.000	18.000 - 64.000	17.000 - 64.000
Income				
- Income less than 1500 monthly	25 (12.0%)	25 (12.0%)	25 (12.0%)	75 (12.0%)
- Income 1500,00 - 1999,99 monthly	54 (26.0%)	54 (26.0%)	54 (26.0%)	162 (26.0%)
- Income 2000,00 - 2499,99 monthly	74 (35.6%)	74 (35.6%)	74 (35.6%)	222 (35.6%)
- Income 2500,00 - 2999,99 monthly	30 (14.4%)	30 (14.4%)	30 (14.4%)	90 (14.4%)
- Income 3000,00 - 4999,99 monthly	7 (3.4%)	7 (3.4%)	7 (3.4%)	21 (3.4%)
- Income $>$ 5000,00 monthly	1 (0.5%)	1 (0.5%)	1 (0.5%)	3 (0.5%)
- Income not stated	17 (8.2%)	17 (8.2%)	17 (8.2%)	51 (8.2%)
Nationality				
- Belgian	207 (99.5%)	207 (99.5%)	207 (99.5%)	621 (99.5%)

Time	Wave 1 (N=208)	Wave 2 (N=208)	Wave 3 (N=208)	Total (N=624)
- Migrant inside EU	1 (0.5%)	1 (0.5%)	1 (0.5%)	3 (0.5%)
- Migrant outside EU	0 (0.0%)	0 (0.0%)	0 (0.0%)	0 (0.0%)
Education				
- No degree	0 (0.0%)	0 (0.0%)	0 (0.0%)	0 (0.0%)
- Primary education	5 (2.4%)	5 (2.4%)	5 (2.4%)	15 (2.4%)
- Secondary education	38 (18.3%)	38 (18.3%)	38 (18.3%)	114 (18.3%)
- Higher education	165 (79.3%)	165 (79.3%)	165 (79.3%)	495 (79.3%)
- Other education	0 (0.0%)	0 (0.0%)	0 (0.0%)	0 (0.0%)
Household				
- Single	25 (12.0%)	25 (12.0%)	25 (12.0%)	75 (12.0%)
- Single with child(ren)	17 (8.2%)	17 (8.2%)	17 (8.2%)	51 (8.2%)
- Couple with child(ren)	138 (66.3%)	138 (66.3%)	138 (66.3%)	414 (66.3%)
- Couple without child(ren)	27 (13.0%)	27 (13.0%)	27 (13.0%)	81 (13.0%)
- Other	1 (0.5%)	1 (0.5%)	1 (0.5%)	3 (0.5%)
Occupation				
- Missing	1	1	1	3
- Medical Personnel	101 (48.8%)	101 (48.8%)	101 (48.8%)	303 (48.8%)
- Paramedics	9 (4.3%)	9 (4.3%)	9 (4.3%)	27 (4.3%)
- Technicians	21 (10.1%)	21 (10.1%)	21 (10.1%)	63 (10.1%)
- Administrative Personnel	41 (19.8%)	41 (19.8%)	41 (19.8%)	123 (19.8%)
- Management	12 (5.8%)	12 (5.8%)	12 (5.8%)	36 (5.8%)
- Cleaning Staff	6 (2.9%)	6 (2.9%)	6 (2.9%)	18 (2.9%)
- Other	17 (8.2%)	17 (8.2%)	17 (8.2%)	51 (8.2%)
Hospital				
- Hospital A	22 (10.6%)	22 (10.6%)	22 (10.6%)	66 (10.6%)
- Hospital B	71 (34.1%)	71 (34.1%)	71 (34.1%)	213 (34.1%)
- Hospital C	80 (38.5%)	80 (38.5%)	80 (38.5%)	240 (38.5%)
- Hospital D	35 (16.8%)	35 (16.8%)	35 (16.8%)	105 (16.8%)
Group				

Time	Wave 1 (N=208)	Wave 2 (N=208)	Wave 3 (N=208)	Total (N=624)
- Non-random control	93 (44.7%)	93 (44.7%)	93 (44.7%)	279 (44.7%)
- Random control	7 (3.4%)	7 (3.4%)	7 (3.4%)	21 (3.4%)
- Non-random intervention	18 (8.7%)	18 (8.7%)	18 (8.7%)	54 (8.7%)
- Random intervention	90 (43.3%)	90 (43.3%)	90 (43.3%)	270 (43.3%)
Language				
- Dutch	200 (96.2%)	200 (96.2%)	200 (96.2%)	600 (96.2%)
- French	3 (1.4%)	3 (1.4%)	3 (1.4%)	9 (1.4%)
- Other	5 (2.4%)	5 (2.4%)	5 (2.4%)	15 (2.4%)
Absenteeism				
- Missing	1	2	3	6
- Mean (SD)	1.130 (7.055)	1.083 (3.867)	1.049 (4.141)	1.087 (5.221)
- Range	0.000 - 63.000	0.000 - 28.000	0.000 - 30.000	0.000 - 63.000
Absenteeism..28				
- Missing	5	2	5	12
- Mean (SD)	0.192 (1.349)	1.083 (3.867)	0.764 (2.989)	0.681 (2.951)
- Range	0.000 - 14.000	0.000 - 28.000	0.000 - 21.000	0.000 - 28.000
Prescribed.medication.use..last.2.weeks.				
- Missing	0	1	0	1
- No	97 (46.6%)	93 (44.9%)	97 (46.6%)	287 (46.1%)
- Yes	111 (53.4%)	114 (55.1%)	111 (53.4%)	336 (53.9%)
Non.prescribed.medication.use..last.2.weeks.				
- Missing	0	1	0	1
- No	144 (69.2%)	129 (62.3%)	133 (63.9%)	406 (65.2%)
- Yes	64 (30.8%)	78 (37.7%)	75 (36.1%)	217 (34.8%)
Year.of.last.PHS				
- Missing	2	9	21	32
- Never	1 (0.5%)	1 (0.5%)	1 (0.5%)	3 (0.5%)
- 2009 or earlier	0 (0.0%)	3 (1.5%)	0 (0.0%)	3 (0.5%)
-2010	0 (0.0%)	0 (0.0%)	0 (0.0%)	0 (0.0%)

Time	Wave 1 (N=208)	Wave 2 (N=208)	Wave 3 (N=208)	Total (N=624)
-2011	0 (0.0%)	0 (0.0%)	0 (0.0%)	0 (0.0%)
-2012	0 (0.0%)	0 (0.0%)	0 (0.0%)	0 (0.0%)
-2013	0 (0.0%)	0 (0.0%)	0 (0.0%)	0 (0.0%)
-2014	1 (0.5%)	0 (0.0%)	0 (0.0%)	1 (0.2%)
-2015	1 (0.5%)	0 (0.0%)	0 (0.0%)	1 (0.2%)
-2016	2 (1.0%)	2 (1.0%)	2 (1.1%)	6 (1.0%)
-2017	23 (11.2%)	11 (5.5%)	8 (4.3%)	42 (7.1%)
-2018	90 (43.7%)	72 (36.2%)	35 (18.7%)	197 (33.3%)
-2019	88 (42.7%)	110 (55.3%)	82 (43.9%)	280 (47.3%)
-2020	0 (0.0%)	0 (0.0%)	59 (31.6%)	59 (10.0%)
Time.since.last.PMO				
- Missing	3	10	22	35
0	88 (42.9%)	0 (0.0%)	59 (31.7%)	147 (25.0%)
-1	90 (43.9%)	110 (55.6%)	82 (44.1%)	282 (47.9%)
-2	23 (11.2%)	72 (36.4%)	35 (18.8%)	130 (22.1%)
-3	2 (1.0%)	11 (5.6%)	8 (4.3%)	21 (3.6%)
-4	1 (0.5%)	2 (1.0%)	2 (1.1%)	5 (0.8%)
-5	1 (0.5%)	0 (0.0%)	0 (0.0%)	1 (0.2%)
-7	0 (0.0%)	0 (0.0%)	0 (0.0%)	0 (0.0%)
-10	0 (0.0%)	0 (0.0%)	0 (0.0%)	0 (0.0%)
-11	0 (0.0%)	3 (1.5%)	0 (0.0%)	3 (0.5%)
Turnover.intention				
- Missing	0	1	0	1
- Mean (SD)	0.846 (1.106)	0.928 (1.242)	0.678 (1.006)	0.817 (1.125)
- Range	0.000 - 4.000	0.000 - 4.000	0.000 - 4.000	0.000 - 4.000
Relation.with.OP				
- I did not yet meet the OP	11 (5.3%)	7 (3.4%)	8 (3.8%)	26 (4.2%)
- < 1 year	29 (13.9%)	16 (7.7%)	14 (6.7%)	59 (9.5%)
- 1 - 2 years	41 (19.7%)	47 (22.6%)	41 (19.7%)	129 (20.7%)

Time	Wave 1 (N=208)	Wave 2 (N=208)	Wave 3 (N=208)	Total (N=624)
- 3 - 4 years	41 (19.7%)	50 (24.0%)	45 (21.6%)	136 (21.8%)
- 5 years or more	86 (41.3%)	88 (42.3%)	100 (48.1%)	274 (43.9%)
Work.related.HC				
- Mean (SD)	1.202 (2.028)	1.231 (2.068)	0.957 (1.853)	1.130 (1.986)
- Range	0.000 - 6.000	0.000 - 6.000	0.000 - 6.000	0.000 - 6.000
Referrals.by.OP.to.other.HC				
- Missing	0	0	1	1
- Mean (SD)	0.202 (0.883)	0.144 (0.721)	0.198 (0.947)	0.181 (0.855)
- Range	0.000 - 6.000	0.000 - 6.000	0.000 - 6.000	0.000 - 6.000
Spontaneous.consultations.with.OP				
- Mean (SD)	0.188 (0.735)	0.149 (0.575)	0.168 (0.692)	0.168 (0.670)
- Range	0.000 - 6.000	0.000 - 6.000	0.000 - 6.000	0.000 - 6.000
Health.Literacy.Score				
- Missing	4	1	4	9
- Mean (SD)	76.884 (10.825)	75.853 (9.335)	77.788 (10.828)	76.837 (10.362)
- Range	37.500 - 100.000	39.062 - 100.000	45.312 - 100.000	37.500 - 100.000
Trust.in.Physician.Score				
- Missing	13	8	8	29
- Mean (SD)	37.554 (6.544)	37.700 (6.439)	38.740 (6.773)	38.002 (6.597)
- Range	14.000 - 55.000	13.000 - 55.000	11.000 - 55.000	11.000 - 55.000
Stress.score				
- Mean (SD)	3.635 (2.353)	3.995 (2.340)	3.966 (2.343)	3.865 (2.347)
- Range	0.000 - 10.000	0.000 - 9.000	0.000 - 10.000	0.000 - 10.000
General.Mental.Health				
- Mean (SD)	1.692 (2.601)	1.986 (2.913)	2.260 (3.221)	1.979 (2.927)
- Range	0.000 - 10.000	0.000 - 12.000	0.000 - 12.000	0.000 - 12.000
Need.for.Recovery				
- Mean (SD)	3.553 (2.764)	3.606 (2.693)	3.615 (2.785)	3.591 (2.743)
- Range	0.000 - 10.000	0.000 - 10.000	0.000 - 11.000	0.000 - 11.000

Time	Wave 1 (N=208)	Wave 2 (N=208)	Wave 3 (N=208)	Total (N=624)
Sleep.score				
- Mean (SD)	4.885 (3.672)	4.817 (3.437)	5.101 (3.593)	4.934 (3.565)
- Range	0.000 - 16.000	0.000 - 16.000	0.000 - 16.000	0.000 - 16.000
Role.conflicts				
- Mean (SD)	3.529 (2.582)	3.562 (2.490)	3.433 (2.460)	3.508 (2.508)
- Range	0.000 - 12.000	0.000 - 10.000	0.000 - 11.000	0.000 - 12.000
Job.satisfaction				
- Mean (SD)	15.284 (2.229)	15.115 (2.415)	15.149 (2.337)	15.183 (2.326)
- Range	8.000 - 20.000	7.000 - 20.000	7.000 - 20.000	7.000 - 20.000
Musculoskeletal.Functioning				
- Mean (SD)	0.981 (1.588)	0.909 (1.509)	2.178 (2.152)	1.356 (1.863)
- Range	0.000 - 9.000	0.000 - 9.000	0.000 - 9.000	0.000 - 9.000

1. Pearson's Chi-squared test

2. Kruskal-Wallis rank sum test

3. Trend test for ordinal variables

Supplement C: descriptive statistics by group

Gender, age, income, nationality, and household are not significantly different in the three waves. In wave 1, the intervention group has slightly more employees with a higher educational attainment, and more medical personnel in comparison with the control group. Most of the medical personnel are nurses: in wave 1 there were 158 nurses in the control group (41.7% of 396), and 188 (50.8% of 380) in the intervention group. These differences are no longer significant in wave 2 or 3.

Supplemental Table S3. Summary Statistics by group (Wave 1) : mean (proportion)

	**control (N=396)**	**intervention (N=380)**	**Total (N=776)**	**p value**
Gender				0.352[1]
Missing	1	1	2	
Male	78 (19.7%)	65 (17.2%)	143 (18.5%)	
Female	317 (80.3%)	314 (82.8%)	631 (81.5%)	
Other	0 (0.0%)	0 (0.0%)	0 (0.0%)	
Age				0.535[2]
Mean (SD)	45.634 (11.215)	45.129 (11.188)	45.387 (11.197)	
Range	22.000 - 66.000	17.000 - 64.000	17.000 - 66.000	
Income				0.801[3]
Missing	3	2	5	
Income less than 1500 monthly	69 (17.6%)	54 (14.3%)	123 (16.0%)	
Income 1500,00 - 1999,99 monthly	117 (29.8%)	123 (32.5%)	240 (31.1%)	
Income 2000,00 - 2499,99 monthly	124 (31.6%)	117 (31.0%)	241 (31.3%)	
Income 2500,00 - 2999,99 monthly	34 (8.7%)	42 (11.1%)	76 (9.9%)	
Income 3000,00 - 4999,99 monthly	15 (3.8%)	11 (2.9%)	26 (3.4%)	
Income > 5000,00 monthly	3 (0.8%)	2 (0.5%)	5 (0.6%)	
Income not stated	31 (7.9%)	29 (7.7%)	60 (7.8%)	
Nationality				0.640[1]
Belgian	388 (98.0%)	375 (98.7%)	763 (98.3%)	
Migrant inside EU	6 (1.5%)	3 (0.8%)	9 (1.2%)	
Migrant outside EU	2 (0.5%)	2 (0.5%)	4 (0.5%)	
Education				0.061[3]

	control (N=396)	intervention (N=380)	Total (N=776)	p value
No degree	3 (0.8%)	4 (1.1%)	7 (0.9%)	
Primary education	14 (3.5%)	9 (2.4%)	23 (3.0%)	
Secondary education	85 (21.5%)	63 (16.6%)	148 (19.1%)	
Higher education	294 (74.2%)	299 (78.7%)	593 (76.4%)	
Other education	0 (0.0%)	5 (1.3%)	5 (0.6%)	
Household				0.640[1]
Single	41 (10.4%)	50 (13.2%)	91 (11.7%)	
Single with child(ren)	34 (8.6%)	29 (7.6%)	63 (8.1%)	
Couple with child(ren)	247 (62.4%)	239 (62.9%)	486 (62.6%)	
Couple without child(ren)	70 (17.7%)	60 (15.8%)	130 (16.8%)	
Other	4 (1.0%)	2 (0.5%)	6 (0.8%)	
Occupation				0.048[1]
Missing	17	10	27	
Medical Personnel	185 (48.8%)	209 (56.5%)	394 (52.6%)	
Paramedics	12 (3.2%)	8 (2.2%)	20 (2.7%)	
Technicians	31 (8.2%)	25 (6.8%)	56 (7.5%)	
Administrative Personnel	55 (14.5%)	65 (17.6%)	120 (16.0%)	
Management	18 (4.7%)	16 (4.3%)	34 (4.5%)	
Cleaning Staff	24 (6.3%)	10 (2.7%)	34 (4.5%)	
Other	54 (14.2%)	37 (10.0%)	91 (12.1%)	
Hospital				0.012[1]
Hospital A	116 (29.3%)	81 (21.3%)	197 (25.4%)	
Hospital B	141 (35.6%)	125 (32.9%)	266 (34.3%)	
Hospital C	93 (23.5%)	121 (31.8%)	214 (27.6%)	
Hospital D	46 (11.6%)	53 (13.9%)	99 (12.8%)	
Group				< 0.001[1]
Non-random control	340 (85.9%)	0 (0.0%)	340 (43.8%)	
Random control	56 (14.1%)	0 (0.0%)	56 (7.2%)	
Non-random intervention	0 (0.0%)	53 (13.9%)	53 (6.8%)	

	control (N=396)	intervention (N=380)	Total (N=776)	p value
Random intervention	0 (0.0%)	327 (86.1%)	327 (42.1%)	

Supplemental Table S4. Summary Statistics by group (Wave 2) : mean (proportion)

	**control (N=202)**	**intervention (N=216)**	**Total (N=418)**	**p value**
Gender				0.638[1]
Missing	8	8	16	
Male	40 (20.6%)	39 (18.8%)	79 (19.7%)	
Female	154 (79.4%)	169 (81.2%)	323 (80.3%)	
Other	0 (0.0%)	0 (0.0%)	0 (0.0%)	
Age				0.842[2]
Missing	7	8	15	
Mean (SD)	46.949 (11.087)	46.577 (11.333)	46.757 (11.203)	
Range	23.000 - 65.000	18.000 - 64.000	18.000 - 65.000	
Income				0.527[3]
Missing	1	0	1	
Income less than 1500 monthly	23 (11.4%)	24 (11.1%)	47 (11.3%)	
Income 1500,00 - 1999,99 monthly	63 (31.3%)	64 (29.6%)	127 (30.5%)	
Income 2000,00 - 2499,99 monthly	58 (28.9%)	69 (31.9%)	127 (30.5%)	
Income 2500,00 - 2999,99 monthly	19 (9.5%)	29 (13.4%)	48 (11.5%)	
Income 3000,00 - 4999,99 monthly	7 (3.5%)	5 (2.3%)	12 (2.9%)	
Income ＞ 5000,00 monthly	2 (1.0%)	1 (0.5%)	3 (0.7%)	
Income not stated	29 (14.4%)	24 (11.1%)	53 (12.7%)	
Nationality				0.477[1]
Belgian	192 (98.5%)	207 (99.5%)	399 (99.0%)	
Migrant inside EU	2 (1.0%)	1 (0.5%)	3 (0.7%)	
Migrant outside EU	1 (0.5%)	0 (0.0%)	1 (0.2%)	
Education				0.183[3]
Missing	7	8	15	

	control (N=202)	intervention (N=216)	Total (N=418)	p value
No degree	0 (0.0%)	0 (0.0%)	0 (0.0%)	
Primary education	4 (2.1%)	5 (2.4%)	9 (2.2%)	
Secondary education	43 (22.1%)	32 (15.4%)	75 (18.6%)	
Higher education	147 (75.4%)	169 (81.2%)	316 (78.4%)	
Other education	1 (0.5%)	2 (1.0%)	3 (0.7%)	
Household				0.270[1]
Missing	7	8	15	
Single	18 (9.2%)	24 (11.5%)	42 (10.4%)	
Single with child(ren)	18 (9.2%)	8 (3.8%)	26 (6.5%)	
Couple with child(ren)	126 (64.6%)	139 (66.8%)	265 (65.8%)	
Couple without child(ren)	31 (15.9%)	35 (16.8%)	66 (16.4%)	
Other	2 (1.0%)	2 (1.0%)	4 (1.0%)	
Occupation				0.119[1]
Missing	2	2	4	
Medical Personnel	89 (44.5%)	123 (57.5%)	212 (51.2%)	
Paramedics	12 (6.0%)	8 (3.7%)	20 (4.8%)	
Technicians	20 (10.0%)	15 (7.0%)	35 (8.5%)	
Administrative Personnel	40 (20.0%)	35 (16.4%)	75 (18.1%)	
Management	12 (6.0%)	10 (4.7%)	22 (5.3%)	
Cleaning Staff	9 (4.5%)	3 (1.4%)	12 (2.9%)	
Other	18 (9.0%)	20 (9.3%)	38 (9.2%)	
Hospital				0.691[1]
Hospital A	53 (26.2%)	48 (22.2%)	101 (24.2%)	
Hospital B	56 (27.7%)	57 (26.4%)	113 (27.0%)	
Hospital C	58 (28.7%)	67 (31.0%)	125 (29.9%)	
Hospital D	35 (17.3%)	44 (20.4%)	79 (18.9%)	
Group				< 0.001[1]
Non-random control	187 (92.6%)	0 (0.0%)	187 (44.7%)	
Random control	15 (7.4%)	0 (0.0%)	15 (3.6%)	

	control (N=202)	intervention (N=216)	Total (N=418)	p value
Non-random intervention	0 (0.0%)	44 (20.4%)	44 (10.5%)	
Random intervention	0 (0.0%)	172 (79.6%)	172 (41.1%)	

Supplemental Table S5. Summary Statistics by group (Wave 3) : mean (proportion)

	control (N=308)	intervention (N=280)	Total (N=588)	p value
Gender				0.810[1]
Missing	15	12	27	
Male	57 (19.5%)	50 (18.7%)	107 (19.1%)	
Female	236 (80.5%)	218 (81.3%)	454 (80.9%)	
Other	0 (0.0%)	0 (0.0%)	0 (0.0%)	
Age				1.000[2]
Missing	14	12	26	
Mean (SD)	46.789 (10.788)	46.806 (10.595)	46.797 (10.686)	
Range	23.000 - 65.000	18.000 - 64.000	18.000 - 65.000	
Income				0.681[3]
Missing	19	15	34	
Income less than 1500 monthly	41 (14.2%)	39 (14.7%)	80 (14.4%)	
Income 1500,00 - 1999,99 monthly	88 (30.4%)	79 (29.8%)	167 (30.1%)	
Income 2000,00 - 2499,99 monthly	99 (34.3%)	87 (32.8%)	186 (33.6%)	
Income 2500,00 - 2999,99 monthly	28 (9.7%)	34 (12.8%)	62 (11.2%)	
Income 3000,00 - 4999,99 monthly	11 (3.8%)	11 (4.2%)	22 (4.0%)	
Income > 5000,00 monthly	0 (0.0%)	2 (0.8%)	2 (0.4%)	
Income not stated	22 (7.6%)	13 (4.9%)	35 (6.3%)	
Nationality				0.580[1]
Missing	9	8	17	
Belgian	292 (99.3%)	265 (98.9%)	557 (99.1%)	
Migrant inside EU	2 (0.7%)	3 (1.1%)	5 (0.9%)	
Migrant outside EU	0 (0.0%)	0 (0.0%)	0 (0.0%)	

	control (N=308)	intervention (N=280)	Total (N=588)	p value
Education				0.380[3]
Missing	14	13	27	
No degree	1 (0.3%)	0 (0.0%)	1 (0.2%)	
Primary education	7 (2.4%)	10 (3.7%)	17 (3.0%)	
Secondary education	58 (19.7%)	40 (15.0%)	98 (17.5%)	
Higher education	227 (77.2%)	214 (80.1%)	441 (78.6%)	
Other education	1 (0.3%)	3 (1.1%)	4 (0.7%)	
Household				0.298[1]
Missing	14	12	26	
Single	26 (8.8%)	29 (10.8%)	55 (9.8%)	
Single with child(ren)	28 (9.5%)	15 (5.6%)	43 (7.7%)	
Couple with child(ren)	188 (63.9%)	185 (69.0%)	373 (66.4%)	
Couple without child(ren)	50 (17.0%)	38 (14.2%)	88 (15.7%)	
Other	2 (0.7%)	1 (0.4%)	3 (0.5%)	
Occupation				0.203[1]
Missing	1	3	4	
Medical Personnel	157 (51.1%)	162 (58.5%)	319 (54.6%)	
Paramedics	13 (4.2%)	11 (4.0%)	24 (4.1%)	
Technicians	28 (9.1%)	12 (4.3%)	40 (6.8%)	
Administrative Personnel	50 (16.3%)	43 (15.5%)	93 (15.9%)	
Management	13 (4.2%)	14 (5.1%)	27 (4.6%)	
Cleaning Staff	16 (5.2%)	8 (2.9%)	24 (4.1%)	
Other	30 (9.8%)	27 (9.7%)	57 (9.8%)	
Hospital				0.112[1]
Hospital A	101 (32.8%)	74 (26.4%)	175 (29.8%)	
Hospital B	81 (26.3%)	66 (23.6%)	147 (25.0%)	
Hospital C	88 (28.6%)	105 (37.5%)	193 (32.8%)	
Hospital D	38 (12.3%)	35 (12.5%)	73 (12.4%)	
Group				< 0.001[1]

	**control (N=308)**	**intervention (N=280)**	**Total (N=588)**	**p value**
Non-random control	266 (86.4%)	0 (0.0%)	266 (45.2%)	
Random control	42 (13.6%)	0 (0.0%)	42 (7.1%)	
Non-random intervention	0 (0.0%)	35 (12.5%)	35 (6.0%)	
Random intervention	0 (0.0%)	245 (87.5%)	245 (41.7%)	

Supplement D: sample quality

Supplemental Table S6. Comparison of sample proportion estimates in time 1 for hospital B C, and D to population data from hospital C, B, and D

Variable	Population B-C (n=1402)	Sample B-C (n=480)	Population D (n=862)	Sample D (n=99)
Gender				
Missing	2.2%	0.2%	0.0%	0.0%
Male	17.8%	20.0%	19.8%	16.16%
Female	80.0%	79.8%	80.2%	83.83%
Age			44.48(12.20)	47.46(11.15)
Missing	2.2%	0.0%		
<25	4.6%	2.1%		
25 - 34	23.7%	21.0%		
35 - 44	22.5%	21.5%		
45 - 54	25.1%	29.2%		
>55	22.0%	26.3%		

Supplement E: full mixed models, including covariates

Supplemental Table S7. Regressions mixed methods: group + time + interaction, and covariates. For each variable, estimates are on the first line, and 95% confidence intervals are in parentheses. *=p<0.1; **=p<0.05; ***=p<0.01. ICC=between/(between+within) variance. σ^2 = within-group variance; τ_{00} = variance of random intercept ; τ_{11} = variance of random slope; ρ_{01}= correlation between random intercept and slope.

Dependent Var	EQ5D vas	Musculo (log)	Stress	Burnout	Sleep Problems	GHQ(log)	NFR(log)
Range	(0-100)	(1-10)	(0-12)	(0-16)	(0-16)	(1-13)	(1-12)
Intercept	76.76 ***	0.54 ***	2.96 ***	4.07 ***	4.63 ***	0.56 ***	1.12 ***
	(73.13 – 80.38)	(0.36 – 0.73)	(2.26 – 3.66)	(3.04 – 5.10)	(3.59 – 5.67)	(0.34 – 0.79)	(0.94 – 1.30)
Intervention group (ref.=control)	-0.26	-0.06	0.02	0.13	0.36	0.07	0.00
	(-2.01 – 1.49)	(-0.15 – 0.02)	(-0.32 – 0.36)	(-0.36 – 0.63)	(-0.14 – 0.86)	(-0.04 – 0.19)	(-0.09 – 0.09)
Time 2 (ref.=Time1)	-1.72 *	-0.12 ***	0.46 ***	0.59 ***	0.07	0.08	0.04
	(-3.50 – 0.05)	(-0.20 – -0.04)	(0.16 – 0.77)	(0.15 – 1.03)	(-0.34 – 0.48)	(-0.04 – 0.19)	(-0.03 – 0.12)
Time 3	-1.11	0.45 ***	0.48 ***	0.69 ***	0.53 **	0.17 ***	0.08 **
	(-2.80 – 0.57)	(0.36 – 0.54)	(0.19 – 0.77)	(0.25 – 1.13)	(0.08 – 0.98)	(0.05 – 0.28)	(0.00 – 0.15)
Hospital B (ref. = hosp A)	3.31 ***	-0.08	-0.38 **	-0.81 ***	-0.82 ***	-0.23 ***	-0.11 **
	(1.41 – 5.21)	(-0.17 – 0.02)	(-0.74 – -0.01)	(-1.35 – -0.27)	(-1.37 – -0.27)	(-0.35 – -0.11)	(-0.21 – -0.02)
Hospital C	2.89 ***	-0.11 **	-0.16	-0.41	-0.49 *	-0.11 *	-0.06
	(0.96 – 4.83)	(-0.20 – -0.01)	(-0.53 – 0.21)	(-0.96 – 0.13)	(-1.05 – 0.07)	(-0.23 – 0.01)	(-0.16 – 0.03)
Hospital D	3.28 ***	-0.14 **	-0.32	-0.42	0.02	-0.07	-0.03
	(0.90 – 5.65)	(-0.26 – -0.02)	(-0.77 – 0.13)	(-1.08 – 0.25)	(-0.66 – 0.70)	(-0.21 – 0.08)	(-0.15 – 0.08)
Age <25 (ref. = Age > 55)	-1.34	-0.07	0.11	0.46	-0.08	-0.11	0.06
	(-6.16 – 3.47)	(-0.31 – 0.17)	(-0.81 – 1.03)	(-0.89 – 1.81)	(-1.42 – 1.27)	(-0.41 – 0.20)	(-0.17 – 0.30)
Age 25 - 34	0.10	-0.05	1.05 ***	1.43 ***	-0.40	0.29 ***	0.13 **
	(-2.09 – 2.29)	(-0.16 – 0.06)	(0.63 – 1.47)	(0.81 – 2.05)	(-1.02 – 0.23)	(0.15 – 0.42)	(0.03 – 0.24)
Age 35 - 44	1.23	-0.05	0.54 ***	0.76 **	0.04	0.08	0.10 **
	(-0.83 – 3.29)	(-0.15 – 0.05)	(0.14 – 0.93)	(0.18 – 1.34)	(-0.54 – 0.63)	(-0.05 – 0.21)	(0.00 – 0.20)
Age 45 - 54	0.14	0.01	0.18	0.43	0.20	0.06	0.06
	(-1.71 – 1.98)	(-0.08 – 0.10)	(-0.17 – 0.53)	(-0.09 – 0.95)	(-0.32 – 0.72)	(-0.06 – 0.17)	(-0.03 – 0.15)
Female (ref. = Male)	-0.99	0.18 ***	0.50 ***	0.93 ***	0.66 **	0.10	0.08
	(-2.86 – 0.89)	(0.08 – 0.27)	(0.14 – 0.86)	(0.40 – 1.47)	(0.12 – 1.20)	(-0.02 – 0.21)	(-0.02 – 0.17)

Dependent Var	EQ5D vas	Musculo (log)	Stress	Burnout	Sleep Problems	GHQ(log)	NFR(log)
Education (linear)	0.07	-0.12	1.50 *	0.30	1.39	-0.50 *	0.20
	(-8.14 – 8.28)	(-0.53 – 0.29)	(-0.09 – 3.08)	(-2.04 – 2.63)	(-0.98 – 3.75)	(-1.01 – 0.02)	(-0.21 – 0.61)
Education (quadratic)	4.00	-0.14	-1.42 **	-2.47 **	-1.47	-0.40 *	-0.42 **
	(-2.99 – 10.99)	(-0.49 – 0.21)	(-2.77 – -0.07)	(-4.46 – -0.49)	(-3.49 – 0.54)	(-0.84 – 0.04)	(-0.76 – -0.07)
Education (cubic)	-6.06 **	0.09	0.99 **	0.57	1.58 **	-0.08	-0.01
	(-10.89 – -1.22)	(-0.15 – 0.33)	(0.06 – 1.92)	(-0.81 – 1.94)	(0.19 – 2.97)	(-0.39 – 0.22)	(-0.25 – 0.23)
Education (4th degree)	3.14 **	0.02	-0.62 **	-0.91 **	-0.81 **	-0.19 **	-0.02
	(0.36 – 5.91)	(-0.12 – 0.16)	(-1.16 – -0.09)	(-1.70 – -0.13)	(-1.60 – -0.01)	(-0.36 – -0.01)	(-0.15 – 0.12)
intervention:time2	-0.46	0.13 **	-0.29	-0.25	-0.27	-0.10	-0.08
	(-2.94 – 2.01)	(0.02 – 0.24)	(-0.72 – 0.14)	(-0.87 – 0.36)	(-0.84 – 0.30)	(-0.26 – 0.06)	(-0.18 – 0.03)
intervention:time3	0.00	0.01	-0.21	-0.57 *	-0.53	-0.15 *	-0.09 *
	(-2.41 – 2.41)	(-0.12 – 0.14)	(-0.62 – 0.20)	(-1.20 – 0.06)	(-1.18 – 0.11)	(-0.32 – 0.01)	(-0.20 – 0.02)
Random Effects							
σ2	13.75	0.03	0.43	0.94	0.91	0.06	0.03
τ00	144.97 ID	0.36 ID	5.73 ID	12.10 ID	12.30 ID	0.60 ID	0.37 ID
τ11	134.63 ID.time2	0.28 ID.time2	4.23 ID.time2	8.43 ID.time2	6.75 ID.time2	0.64 ID.time2	0.23 ID.time2
	173.98 ID.time3	0.50 ID.time3	4.66 ID.time3	11.21 ID.time3	11.83 ID.time3	0.90 ID.time3	0.31 ID.time3
ρ01	-0.33	-0.45	-0.44	-0.43	-0.39	-0.5	-0.45
	-0.51	-0.47	-0.49	-0.47	-0.46	-0.57	-0.44
ICC	0.92	0.92	0.93	0.93	0.93	0.91	0.93
N	1041 ID	1042 ID	1042 ID	1042 ID	1042 ID	1042 ID	1042 ID
Observations	1733	1736	1736	1736	1736	1736	1736
Marginal R2 / Conditional R2	0.029 / 0.923	0.132 / 0.932	0.048 / 0.932	0.046 / 0.931	0.025 / 0.934	0.037 / 0.916	0.023 / 0.930

Supplemental Table S8. Regressions mixed methods: group + time + interaction, and covariates. For each variable, estimates are on the first line, and 95% confidence intervals are in parentheses. *=p<0.1; **=p<0.05; ***=p<0.01. ICC=between/(between+within) variance. σ^2 = within-group variance; τ00 = variance of random intercept ; τ11 = variance of random slope; ρ01= correlation between random intercept and slope.

Dependent Var	Role conflicts	Health literacy	Trust in physician	Job Satisfaction	Turnover intention
Range	(0-12)	(0-100)	(0-55)	(0-20)	(0-4)

Dependent Var	Role conflicts	Health literacy	Trust in physician	Job Satisfaction	Turnover intention
Intercept	3.65 ***	76.25 ***	40.59 ***	15.53 ***	0.37 **
	(2.95 – 4.36)	(73.27 – 79.23)	(38.85 – 42.33)	(14.86 – 16.20)	(0.05 – 0.68)
Intervention group (ref.=control)	0.30 *	-0.97	-0.94 **	-0.07	-0.02
	(-0.04 – 0.64)	(-2.43 – 0.49)	(-1.78 – -0.10)	(-0.38 – 0.25)	(-0.17 – 0.13)
Time 2 (ref.=Time1)	0.16	-1.31 *	-0.32	-0.09	0.19 ***
	(-0.13 – 0.46)	(-2.62 – -0.00)	(-0.99 – 0.36)	(-0.35 – 0.17)	(0.05 – 0.32)
Time 3	0.13	1.22 *	-0.04	-0.06	-0.15 **
	(-0.17 – 0.43)	(-0.19 – 2.63)	(-0.79 – 0.72)	(-0.33 – 0.21)	(-0.28 – -0.02)
Hospital B (ref. = hosp A)	-0.79 ***	0.49	-0.16	0.31 *	-0.16 *
	(-1.16 – -0.42)	(-1.10 – 2.07)	(-1.09 – 0.77)	(-0.04 – 0.67)	(-0.33 – 0.00)
Hospital C	-0.02	-0.03	0.53	0.14	0.19 **
	(-0.39 – 0.36)	(-1.63 – 1.57)	(-0.40 – 1.47)	(-0.22 – 0.50)	(0.02 – 0.35)
Hospital D	-0.20	-3.36 ***	-4.77 ***	0.24	-0.03
	(-0.66 – 0.25)	(-5.31 – -1.41)	(-5.89 – -3.64)	(-0.20 – 0.68)	(-0.24 – 0.18)
Age <25 (ref. = Age > 55)	0.91 *	-5.44 ***	-2.55 **	0.34	0.47 **
	(-0.01 – 1.83)	(-9.45 – -1.43)	(-4.83 – -0.28)	(-0.51 – 1.20)	(0.05 – 0.89)
Age 25 - 34	0.99 ***	-1.29	-0.98 *	-0.44 **	0.84 ***
	(0.57 – 1.41)	(-3.08 – 0.50)	(-2.03 – 0.07)	(-0.85 – -0.04)	(0.65 – 1.03)
Age 35 - 44	0.63 ***	-0.55	-0.55	-0.26	0.65 ***
	(0.23 – 1.03)	(-2.26 – 1.15)	(-1.54 – 0.44)	(-0.63 – 0.12)	(0.47 – 0.83)
Age 45 - 54	-0.11	-0.11	0.08	-0.28 *	0.48 ***
	(-0.46 – 0.24)	(-1.63 – 1.41)	(-0.79 – 0.95)	(-0.61 – 0.05)	(0.32 – 0.64)
Female (ref. = Male)	-0.46 **	-0.74	-0.69	0.53 ***	-0.23 ***
	(-0.82 – -0.09)	(-2.28 – 0.80)	(-1.60 – 0.22)	(0.18 – 0.88)	(-0.40 – -0.07)
Education (linear)	-1.68 **	1.60	1.43	0.55	-0.23
	(-3.28 – -0.07)	(-5.16 – 8.35)	(-2.51 – 5.37)	(-0.97 – 2.08)	(-0.95 – 0.49)
Education (quadratic)	-0.10	1.27	1.75	0.83	-0.54 *
	(-1.47 – 1.26)	(-4.48 – 7.03)	(-1.61 – 5.11)	(-0.47 – 2.13)	(-1.15 – 0.07)
Education (cubic)	-1.01 **	-0.65	1.81	1.16 **	-0.42 *
	(-1.95 – -0.07)	(-4.64 – 3.35)	(-0.52 – 4.13)	(0.26 – 2.05)	(-0.84 – 0.01)
Education (4th degree)	-0.08	0.15	-0.01	-0.10	0.01

Dependent Var	Role conflicts	Health literacy	Trust in physician	Job Satisfaction	Turnover intention
	(-0.62 – 0.46)	(-2.16 – 2.46)	(-1.35 – 1.33)	(-0.62 – 0.41)	(-0.23 – 0.26)
intervention:time2	-0.27	1.55 *	0.32	0.08	-0.18 *
	(-0.68 – 0.14)	(-0.27 – 3.38)	(-0.61 – 1.25)	(-0.28 – 0.44)	(-0.37 – 0.00)
intervention:time3	-0.22	-0.36	0.76	0.05	0.15
	(-0.65 – 0.22)	(-2.38 – 1.65)	(-0.32 – 1.85)	(-0.33 – 0.43)	(-0.04 – 0.34)
Random Effects					
σ2	0.44	8.57	2.39	0.36	0.09
τ00	5.70 ID	99.95 ID	33.41 ID	5.13 ID	1.11 ID
τ11	3.60 ID.time2	72.66 ID.time2	15.86 ID.time2	2.48 ID.time2	0.70 ID.time2
	5.22 ID.time3	113.80 ID.time3	31.01 ID.time3	3.86 ID.time3	0.95 ID.time3
ρ01	-0.43	-0.49	-0.36	-0.28	-0.29
	-0.46	-0.45	-0.45	-0.46	-0.45
ICC	0.93	0.92	0.93	0.93	0.93
N	1042 ID	1017 ID	1001 ID	1040 ID	1034 ID
Observations	1736	1688	1642	1734	1723
Marginal R2 / Conditional R2	0.058 / 0.932	0.027 / 0.926	0.094 / 0.940	0.024 / 0.936	0.098 / 0.938

Supplemental Table S9. Regressions mixed methods: group + time + interaction, and covariates. For each variable, estimates are on the first line, and 95% confidence intervals are in parentheses. *=p<0.1; **=p<0.05; ***=p<0.01. ICC=between/(between+within) variance. σ^2 = within-group variance; τ00 = variance of random intercept ; τ11 = variance of random slope; ρ01= correlation between random intercept and slope.

Dependent Var	Generalised Poisson absenteeism	Generalised Poisson absenteeism last 4 weeks	Binomial work-related consultations	Negative binomial referrals	Negative binomial spontaneous consultations	Binomial prescribed medication	Binomial non-prescribed medication
Predictors	Incidence Rate Ratios	Incidence Rate Ratios	Odds Ratios	Incidence Rate Ratios	Incidence Rate Ratios	Odds Ratios	Odds Ratios
Intercept	2.23 *	5.12 ***	0.53 **	0.64	0.39 ***	2.49 **	0.14 ***
	(0.93 – 5.35)	(1.93 – 13.54)	(0.31 – 0.89)	(0.05 – 8.44)	(0.24 – 0.64)	(1.19 – 5.20)	(0.07 – 0.24)
Intervention group (ref.=control)	0.59 **	0.30 ***	0.69 **	0.46 ***	0.35 ***	1.53 *	1.23
	(0.37 – 0.95)	(0.14 – 0.63)	(0.47 – 1.00)	(0.28 – 0.76)	(0.23 – 0.55)	(0.93 – 2.50)	(0.83 – 1.82)

Dependent Var	Generalised Poisson absenteeism	Generalised Poisson absenteeism last 4 weeks	Binomial work-related consultations	Negative binomial referrals	Negative binomial spontaneous consultations	Binomial prescribed medication	Binomial non-prescribed medication
Time 2 (ref.=Time1)	1.45	1.36	0.80	0.58 *	0.54 ***	1.30	1.51 *
	(0.91 – 2.29)	(0.74 – 2.51)	(0.52 – 1.23)	(0.33 – 1.00)	(0.34 – 0.86)	(0.78 – 2.17)	(0.97 – 2.34)
Time 3	0.83	1.38	0.62 **	0.48 ***	0.59 ***	0.88	1.32
	(0.52 – 1.33)	(0.74 – 2.59)	(0.43 – 0.92)	(0.29 – 0.80)	(0.40 – 0.87)	(0.56 – 1.40)	(0.89 – 1.96)
Hospital B (ref. = hosp A)	0.85	1.75 ***	0.64 **	0.88	0.79	0.94	0.85
	(0.59 – 1.23)	(1.16 – 2.64)	(0.45 – 0.92)	(0.55 – 1.42)	(0.57 – 1.11)	(0.57 – 1.56)	(0.58 – 1.23)
Hospital C	0.78	0.78	0.79	1.13	0.82	0.59 **	1.01
	(0.54 – 1.13)	(0.49 – 1.23)	(0.55 – 1.14)	(0.74 – 1.74)	(0.57 – 1.16)	(0.35 – 0.99)	(0.70 – 1.47)
Hospital D	0.46 ***	0.75	0.80	0.74	0.58 **	0.65	0.96
	(0.27 – 0.80)	(0.30 – 1.86)	(0.51 – 1.25)	(0.40 – 1.34)	(0.36 – 0.95)	(0.34 – 1.23)	(0.61 – 1.51)
Age <25 (ref. = Age > 55)	0.90	0.53	0.87	1.67	1.95 *	0.14 ***	2.04
	(0.28 – 2.89)	(0.14 – 2.00)	(0.33 – 2.28)	(0.61 – 4.62)	(0.91 – 4.15)	(0.04 – 0.55)	(0.77 – 5.42)
Age 25 - 34	1.45 *	0.97	0.99	0.58 **	0.97	0.18 ***	1.07
	(0.96 – 2.21)	(0.59 – 1.59)	(0.66 – 1.50)	(0.34 – 0.99)	(0.65 – 1.45)	(0.09 – 0.34)	(0.70 – 1.65)
Age 35 - 44	1.06	1.47	0.80	0.75	1.00	0.20 ***	1.52 **
	(0.69 – 1.61)	(0.86 – 2.52)	(0.54 – 1.18)	(0.47 – 1.20)	(0.69 – 1.46)	(0.11 – 0.36)	(1.02 – 2.27)
Age 45 - 54	0.99	1.37	0.98	0.91	0.79	0.43 ***	1.09
	(0.67 – 1.47)	(0.88 – 2.14)	(0.69 – 1.40)	(0.61 – 1.37)	(0.55 – 1.12)	(0.26 – 0.72)	(0.75 – 1.57)
Female (ref. = Male)	1.83 ***	1.03	1.97 ***	1.24	1.32	1.29	1.87 ***
	(1.17 – 2.87)	(0.47 – 2.25)	(1.36 – 2.84)	(0.80 – 1.92)	(0.91 – 1.92)	(0.78 – 2.14)	(1.28 – 2.72)
Education (linear)	0.78	0.54 ***	1.16	0.54 ***	0.73	0.93	1.78 **
	(0.50 – 1.24)	(0.35 – 0.84)	(0.71 – 1.89)	(0.34 – 0.83)	(0.48 – 1.11)	(0.47 – 1.84)	(1.04 – 3.04)
	1.01	1.03	1.01	1.17	0.90	0.90	1.03

Dependent Var	Generalised Poisson absenteeism	Generalised Poisson absenteeism last 4 weeks	Binomial work-related consultations	Negative binomial referrals	Negative binomial spontaneous consultations	Binomial prescribed medication	Binomial non-prescribed medication
Education (quadratic)	(0.70 – 1.47)	(0.69 – 1.53)	(0.69 – 1.48)	(0.80 – 1.70)	(0.65 – 1.25)	(0.53 – 1.54)	(0.68 – 1.55)
intervention:time 2	1.38	4.11 ***	1.20	1.16	2.51 **	0.56	0.97
	(0.68 – 2.79)	(1.69 – 10.01)	(0.66 – 2.18)	(0.46 – 2.88)	(1.20 – 5.27)	(0.28 – 1.16)	(0.53 – 1.79)
intervention:time 3	1.91 *	2.96 **	1.73 *	1.78	2.47 ***	0.95	1.27
	(0.94 – 3.85)	(1.11 – 7.89)	(1.00 – 2.99)	(0.80 – 3.97)	(1.29 – 4.75)	(0.49 – 1.83)	(0.73 – 2.21)
Zero-Inflated Model							
Intercept		7.19 ***		0.24			
Dispersion Model							
overdispersion parameter	521	7.19		3.5	1.11		
Random Effects							
σ2			3.29			3.29	3.29
τ00			1.37 ID			4.30 ID	1.44 ID
ICC			0.29			0.57	0.31
N			1042 ID			1042 ID	1042 ID
Observations	1714	1684	1735	1734	1736	1734	1734
R2 conditional / R2 marginal	NA / 0.410	NA / 0.344	0.028 / 0.313	NA / 0.077	NA / 0.084	0.073 / 0.598	0.046 / 0.337

Supplemental Table S10. Regressions mixed methods: group + time + interaction, and covariates. For each variable, estimates are on the first line, and 95% confidence intervals are in parentheses. *=p<0.1; **=p<0.05; ***=p<0.01. ICC=between/(between+within) variance. σ^2 = within-group variance; τ00 = variance of random intercept ; τ11 = variance of random slope; ρ01= correlation between random intercept and slope.

Dependent Var	Weighted Presenteeism		
Range	(0-140)		
Predictors	Incidence Rate Ratios		
Intercept	20.30 ***	Dispersion Model	
	(15.47 – 26.63)	Intercept	12.47 ***
Intervention group (ref.=control)	1.15		(7.96 – 19.53)
	(0.95 – 1.39)	Hospital B (ref. = hosp A)	0.93
Time 2 (ref.=Time1)	0.98		(0.56 – 1.55)
	(0.77 – 1.25)	Hospital C	0.77
Time 3	1.07		(0.46 – 1.30)
	(0.87 – 1.31)	Hospital D	1.25
Hospital B (ref. = hosp A)	0.97		(0.66 – 2.36)
	(0.78 – 1.20)	Age <25 (ref. = Age > 55)	3.24
Hospital C	0.83 *		(0.67 – 15.64)
	(0.67 – 1.03)	Age 25 - 34	1.48
Hospital D	1.09		(0.83 – 2.61)
	(0.83 – 1.44)	Age 35 - 44	1.44
Age <25 (ref. = Age > 55)	1.29		(0.84 – 2.44)
	(0.62 – 2.71)	Age 45 - 54	1.80 **
Age 25 - 34	1.04		(1.08 – 3.00)
	(0.82 – 1.33)	Zero-Inflated Model	
Age 35 - 44	1.12	Intercept	1.90 ***
	(0.90 – 1.40)		(1.45 – 2.48)
Age 45 - 54	1.13	Hospital B (ref. = hosp A)	1.73 ***
	(0.91 – 1.40)		(1.31 – 2.29)
Female (ref. = Male)	0.83 **	Hospital C	1.33 **
	(0.70 – 0.99)		(1.01 – 1.75)

Education (linear)	0.88	Hospital D	1.44 **
	(0.71 – 1.08)		(1.02 – 2.02)
Education (quadratic)	0.96	Age <25<br>(ref. = Age > 55)	0.56
	(0.81 – 1.14)		(0.27 – 1.17)
intervention:time2	0.97	Age 25 - 34	0.48 ***
	(0.69 – 1.35)		(0.35 – 0.66)
intervention:time3	0.88	Age 35 - 44	0.69 **
	(0.66 – 1.18)		(0.51 – 0.93)
		Age 45 - 54	0.71 **
			(0.54 – 0.94)
Observations	1638		
R^2 conditional / R^2marginal	NA / 0.026		

Supplemental Table S11. Regressions mixed methods: group + time + interaction, and covariates. For each variable, estimates are on the first line, and 95% confidence intervals are in parentheses. *=p<0.1; **=p<0.05; ***=p<0.01. ICC=between/(between+within) variance. σ^2 = within-group variance; $\tau00$ = variance of random intercept ; $\tau11$ = variance of random slope; $\rho01$= correlation between random intercept and slope.

Dependent Var	Worry intensity	Worry frequency	Worry weighted score
Range	(1-5)	(1-5)	(0-1.55)
Intercept	1.41 ***	1.82 ***	0.72 ***
	(1.19 – 1.64)	(1.51 – 2.14)	(0.61 – 0.83)
Intervention group (ref.=control)	-0.06	-0.03	-0.02
	(-0.17 – 0.06)	(-0.18 – 0.13)	(-0.07 – 0.03)
Time 2 (ref.=Time1)	-0.14 **	0.01	-0.03
	(-0.24 – -0.03)	(-0.12 – 0.15)	(-0.08 – 0.01)
Time 3	-0.06	0.24 ***	0.03

Dependent Var	Worry intensity	Worry frequency	Worry weighted score
	(-0.16 – 0.04)	(0.11 – 0.38)	(-0.01 – 0.08)
Hospital B (ref. = hosp A)	-0.11 *	-0.28 ***	-0.08 ***
	(-0.23 – 0.01)	(-0.44 – -0.11)	(-0.14 – -0.03)
Hospital C	-0.15 **	-0.20 **	-0.08 ***
	(-0.27 – -0.03)	(-0.37 – -0.03)	(-0.14 – -0.02)
Hospital D	-0.13 *	-0.38 ***	-0.11 ***
	(-0.28 – 0.01)	(-0.58 – -0.17)	(-0.18 – -0.04)
Age<25 (ref. = Age > 55)	-0.09	0.11	-0.00
	(-0.38 – 0.21)	(-0.30 – 0.52)	(-0.14 – 0.14)
Age 25 - 34	-0.23 ***	-0.12	-0.08 **
	(-0.37 – -0.10)	(-0.31 – 0.07)	(-0.15 – -0.02)
Age 35 - 44	-0.26 ***	-0.17 *	-0.10 ***
	(-0.39 – -0.14)	(-0.35 – 0.01)	(-0.16 – -0.04)
Age 45 - 54	-0.09	-0.15 *	-0.05 *
	(-0.20 – 0.02)	(-0.31 – 0.01)	(-0.11 – 0.00)
Female (ref. = Male)	0.04	0.15 *	0.04
	(-0.08 – 0.15)	(-0.02 – 0.31)	(-0.02 – 0.09)
Education (linear)	-0.14	-0.40	-0.12
	(-0.65 – 0.37)	(-1.12 – 0.32)	(-0.36 – 0.13)
Education (quadratic)	0.06	0.30	0.07
	(-0.37 – 0.50)	(-0.32 – 0.91)	(-0.14 – 0.28)
Education (cubic)	0.14	-0.16	0.00
	(-0.16 – 0.44)	(-0.58 – 0.27)	(-0.14 – 0.15)
Education (4th degree)	0.03	0.08	0.02
	(-0.14 – 0.20)	(-0.16 – 0.33)	(-0.06 – 0.11)
intervention:time2	0.20 ***	0.10	0.07 **
	(0.05 – 0.35)	(-0.08 – 0.29)	(0.01 – 0.13)
intervention:time3	0.03	-0.07	-0.00
	(-0.11 – 0.18)	(-0.27 – 0.12)	(-0.07 – 0.06)
Random Effects			

Dependent Var	Worry intensity	Worry frequency	Worry weighted score
σ2	0.05	0.09	0.01
τ00	0.62 ID	1.15 ID	0.14 ID
τ11	0.46 ID.time2	0.72 ID.time2	0.08 ID.time2
	0.61 ID.time3	1.07 ID.time3	0.12 ID.time3
ρ01	-0.42	-0.39	-0.36
	-0.57	-0.48	-0.5
ICC	0.92	0.93	0.93
N	1042 ID	1042 ID	1042 ID
Observations	1736	1735	1735
Marginal R2 / Conditional R2	0.037 / 0.922	0.030 / 0.930	0.031 / 0.931

Supplement F: post-hoc estimations

Supplemental Table S12. Posthoc estimations with contrasts of within (one group over time) and between (same time different groups) group with 95% confidence intervals in parentheses

**contrasts**	**c1-i1**	**c2-i2**	**c3-i3**	**c1-c2**	**c2-c3**	**i1-i2**	**i2-i3**
EQ5DVAS	0.261	0.723	0.26	1.724	-0.609	2.186	-1.072
	(-2.297 – 2.82)	(-2.907 – 4.354)	(-2.801 – 3.322)	(-0.874 – 4.321)	(-3.48 – 2.262)	(-0.345 – 4.717)	(-3.875 – 1.731)
NMQ	0.065	-0.066	0.056	0.115 *	-0.566	-0.016	-0.443 ***
	(-0.063 – 0.192)	(-0.226 – 0.093)	(-0.11 – 0.222)	(-0.002 – 0.233)	(-0.705 – - 0.426)	(-0.131 – 0.099)	(-0.581 – - 0.306)
Stress	-0.02	0.27	0.19	-0.462 **	-0.02	-0.172	-0.1
	(-0.517 – 0.476)	(-0.344 – 0.884)	(-0.351 – 0.732)	(-0.912 – - 0.013)	(-0.486 – 0.447)	(-0.611 – 0.268)	(-0.553 – 0.353)
Burnout	-0.134	0.121	0.436	-0.586 *	-0.102	-0.332	0.213
	(-0.858 – 0.591)	(-0.774 – 1.016)	(-0.397 – 1.268)	(-1.232 – 0.059)	(-0.803 – 0.599)	(-0.964 – 0.299)	(-0.471 – 0.897)
Sleep Problems	-0.361	-0.092	0.173	-0.07	-0.455	0.199	-0.191
	(-1.088 – 0.366)	(-0.964 – 0.78)	(-0.681 – 1.026)	(-0.669 – 0.529)	(-1.151 – 0.24)	(-0.387 – 0.785)	(-0.873 – 0.491)
GHQ	-0.075	0.028	0.076	-0.078	-0.091	0.024	-0.042
	(-0.241 – 0.092)	(-0.192 – 0.248)	(-0.124 – 0.277)	(-0.248 – 0.092)	(-0.275 – 0.093)	(-0.143 – 0.191)	(-0.223 – 0.138)
NFR	-0.001	0.077	0.091	-0.041	-0.036	0.036	-0.022
	(-0.126 – 0.125)	(-0.074 – 0.227)	(-0.054 – 0.236)	(-0.15 – 0.067)	(-0.159 – 0.087)	(-0.07 – 0.141)	(-0.143 – 0.099)
HLS	0.97	-0.582	1.333	1.314	-2.532 **	-0.239	-0.617
	(-1.167 – 3.107)	(-3.152 – 1.987)	(-1.321 – 3.987)	(-0.605 – 3.232)	(-4.769 – - 0.295)	(-2.109 – 1.631)	(-2.825 – 1.592)
Trust	0.936	0.617	0.172	0.318	-0.282	-0.001	-0.727
	(-0.293 – 2.166)	(-0.84 – 2.075)	(-1.265 – 1.609)	(-0.668 – 1.305)	(-1.469 – 0.906)	(-0.95 – 0.949)	(-1.9 – 0.446)
Job Satisfaction	0.065	-0.015	0.013	0.086	-0.023	0.005	0.005
	(-0.399 – 0.53)	(-0.603 – 0.573)	(-0.507 – 0.532)	(-0.294 – 0.465)	(-0.48 – 0.435)	(-0.364 – 0.375)	(-0.444 – 0.453)

contrasts	c1-i1	c2-i2	c3-i3	c1-c2	c2-c3	i1-i2	i2-i3
Role conflicts	-0.299	-0.032	-0.082	-0.162	0.033	0.105	-0.018
	(-0.796 – 0.197)	(-0.637 – 0.572)	(-0.658 – 0.493)	(-0.593 – 0.268)	(-0.459 – 0.524)	(-0.316 – 0.525)	(-0.499 – 0.464)
Turnover intention	0.023	0.206	-0.126	-0.187 *	0.335 ***	-0.003	0.003
	(-0.196 – 0.242)	(-0.083 – 0.496)	(-0.378 – 0.126)	(-0.382 – 0.008)	(0.115 – 0.555)	(-0.194 – 0.187)	(-0.213 – 0.218)
Absenteeism (gen poisson)	0.527	0.206	-0.117	-0.37	0.556	-0.691	0.232
	(-0.164 – 1.219)	(-0.551 – 0.964)	(-0.874 – 0.64)	(-1.038 – 0.299)	(-0.194 – 1.306)	(-1.469 – 0.088)	(-0.532 – 0.997)
Absenteeism last 4 weeks (gen poisson)	1.207 **	-0.206	0.122	-0.309	-0.016	-1.723 ***	0.312
	(0.13 – 2.283)	(-0.895 – 0.482)	(-0.79 – 1.034)	(-1.198 – 0.58)	(-0.846 – 0.814)	(-2.759 – -0.686)	(-0.451 – 1.075)
Presenteeism weighted (gen poisson)	-0.138	-0.103	-0.011	0.019	-0.086	0.053	0.007
	(-0.416 – 0.14)	(-0.505 – 0.298)	(-0.337 – 0.315)	(-0.338 – 0.375)	(-0.455 – 0.284)	(-0.284 – 0.39)	(-0.359 – 0.372)
HC Work-related (binom)	0.375	0.193	-0.172	0.222	0.248	0.04	-0.117
	(-0.17 – 0.92)	(-0.554 – 0.94)	(-0.812 – 0.468)	(-0.397 – 0.842)	(-0.418 – 0.914)	(-0.573 – 0.653)	(-0.774 – 0.54)
HC Referrals (nbinom)	0.772 **	0.626	0.195	0.547	0.181	0.401	-0.25
	(0.057 – 1.488)	(-0.535 – 1.787)	(-0.738 – 1.127)	(-0.256 – 1.35)	(-0.752 – 1.115)	(-0.661 – 1.462)	(-1.402 – 0.902)
HC Spontaneous (nbinom)	1.038 ***	0.117	0.132	0.62 *	-0.086	-0.3	-0.071
	(0.411 – 1.664)	(-0.763 – 0.998)	(-0.586 – 0.849)	(-0.065 – 1.305)	(-0.856 – 0.685)	(-1.135 – 0.535)	(-0.906 – 0.764)
Prescribed Medication (binom)	-0.424	0.147	-0.369	-0.261	0.386	0.31	-0.13
	(-1.141 – 0.294)	(-0.8 – 1.095)	(-1.193 – 0.456)	(-1.006 – 0.485)	(-0.414 – 1.186)	(-0.423 – 1.044)	(-0.914 – 0.654)

**contrasts**	**c1-i1**	**c2-i2**	**c3-i3**	**c1-c2**	**c2-c3**	**i1-i2**	**i2-i3**
Non-Prescribed Medication (binom)	-0.206	-0.18	-0.443	-0.41	0.131	-0.384	-0.132
	(-0.774 – 0.361)	(-0.934 – 0.575)	(-1.088 – 0.202)	(-1.05 – 0.229)	(-0.541 – 0.803)	(-1.004 – 0.237)	(-0.788 – 0.525)

Supplemental Figure S1. Predicted effects plots of mixed models with 95% confidence intervals, blue = control group, orange = intervention group. X-axis=measurement moment (1,2,3). The post-hoc linear predicted means of each outcome are based on the regression estimates, with their confidence intervals, for each measurement moment (x-axis) and by group (colour)

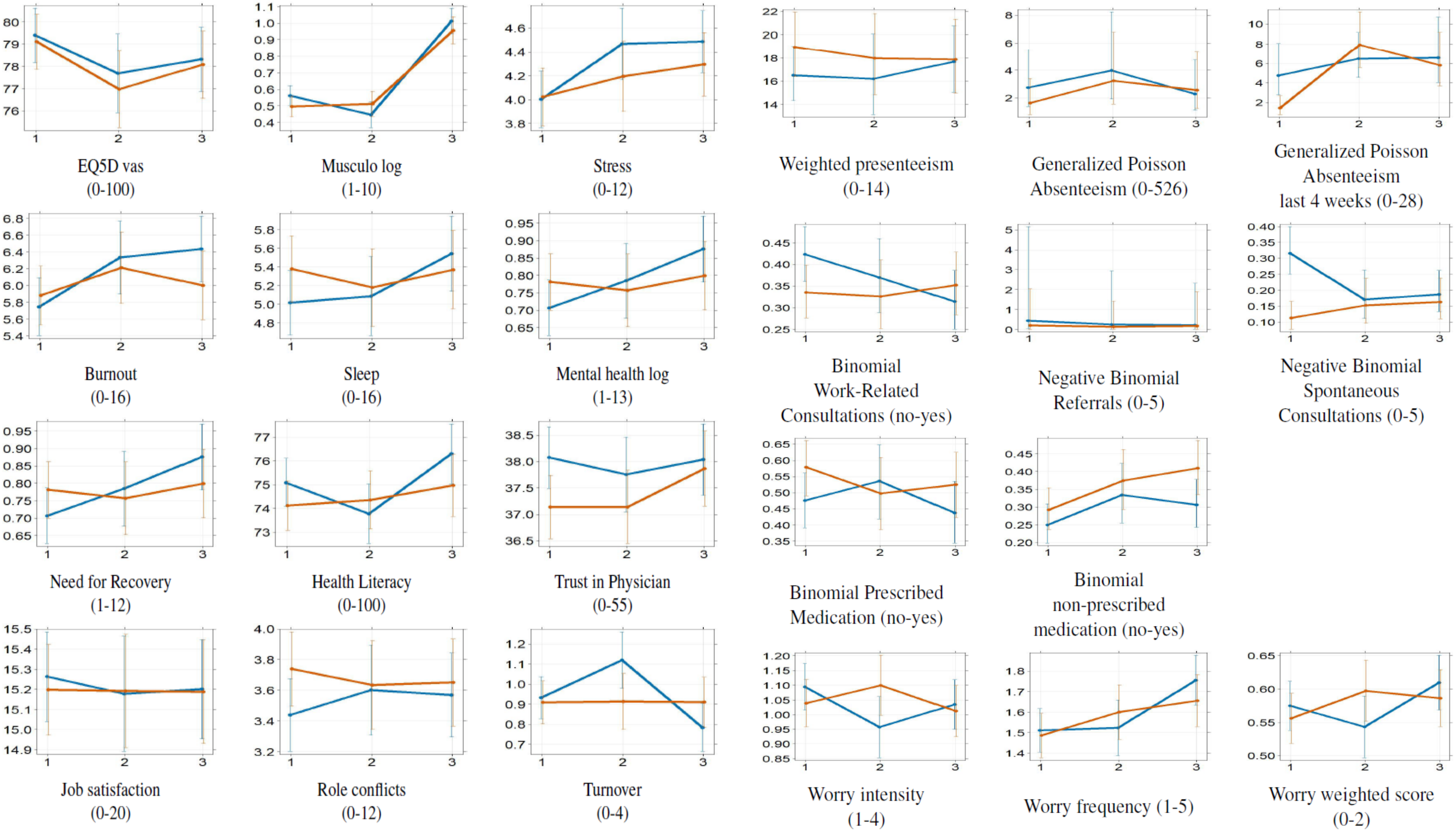

Supplemental Table S13. Posthoc estimations with contrasts of within (one group over time) and between (same time different groups) group, with p-value of non-inferiority and non-superiority tests. The estimates and non-inferiority and non-superiority tests for the post-hoc estimations are in reversed direction (intervention – control) and the relevant differences (delta) above or below which non-inferiority or non-superiority applies are stated. The estimates for which there is no indication for non-inferiority or non-superiority are formatted in bold. For each variable, estimates are on the first line, and p-values on the second.

contrasts	delta	direction	i1-c1	i2-c2	i3-c3	i2-i1	i3-i2	c2-c1	c3-c2
EQ5DVAS	5	noninferior	-0.261 **	-0.723 **	-0.26 **	-2.186 **	1.072 **	-1.724 **	0.609 **
			0	0.006	0.0001	0.012	0	0.003	0
NMQ	log(0.45)	nonsuperior	-0.065 **	0.066 **	-0.056 **	0.016 **	0.443 **	-0.115 **	0.566 **
			0	0	0	0	0	0	0.00002
Stress	0.6	nonsuperior	0.02 **	-0.27 **	-0.19 **	0.172 *	0.1 **	0.462	0.02 **
			0.007	0.0004	0.0003	0.041	0.013	0.959	0.003
Burnout	0.8	nonsuperior	0.134	-0.121 *	-0.436 **	0.332	-0.213 **	0.586	0.102 *
			0.064	0.025	0.0002	0.23	0.0002	0.941	0.034
Sleep Problems	0.8	nonsuperior	0.361	0.092	-0.173 **	-0.199 **	0.191	0.07 **	0.455
			0.478	0.144	0.009	0.00001	0.079	0.004	0.707
GHQ	log(0.6)	nonsuperior	0.075 **	-0.028 **	-0.076 **	-0.024 **	0.042 **	0.078 **	0.091 **
			0	0	0	0	0	0	0
NFR	log(0.6)	nonsuperior	0.001 **	-0.077 **	-0.091 **	-0.036 **	0.022 **	0.041 **	0.036 **
			0	0	0	0	0	0	0
HLS	5	noninferior	-0.97 **	0.582 **	-1.333 **	0.239 **	0.617 **	-1.314 **	2.532 **
			0	0.00001	0	0	0	0	0.013
Trust	2.75	noninferior	-0.936 **	-0.617 **	-0.172 **	0.001 **	0.727 **	-0.318 **	0.282 **
			0.0002	0.0002	0	0	0	0	0

Job Satisfaction	1	noninferior	-0.065 **	0.015 **	-0.013 **	-0.005 **	-0.005 **	-0.086 **	0.023 **
			0	0.00001	0	0	0	0	0
Role conflicts	0.6	nonsuperior	0.299	0.032 *	0.082	-0.105 **	0.018 **	0.162 *	-0.033 **
			0.474	0.054	0.075	0.00002	0.004	0.028	0.002
Turnover intention	0.2	nonsuperior	-0.023 *	-0.206 **	0.126	0.003 *	-0.003 *	0.187	-0.335 **
			0.028	0.0005	0.965	0.025	0.053	1	0
Absenteeism (gen poisson)	1	nonsuperior	-0.527 **	-0.206 **	0.117 **	0.691	-0.232 **	0.37 *	-0.556 **
			0	0.00004	0.007	0.873	0.00003	0.053	0
Absenteeism last 4 weeks (gen poisson)	1	nonsuperior	-1.207 **	0.206 **	-0.122 **	1.723	-0.312 **	0.309	0.016 **
			0	0.008	0.003	1	0.00001	0.183	0.006
HC Work-related (binom)	1	nonsuperior	-0.375 **	-0.193 **	0.172 **	-0.04 **	0.117 **	-0.222 **	-0.248 **
			0	0.00004	0.002	0.00001	0.001	0	0
HC Referrals (nbinom)	1	nonsuperior	-0.772 **	-0.626 **	-0.195 **	-0.401 **	0.25	-0.547 **	-0.181 **
			0	0.001	0.002	0.001	0.383	0	0.002
HC Spontaneous (nbinom)	1	nonsuperior	-1.038 **	-0.117 **	-0.132 **	0.3	0.071 **	-0.62 **	0.086 **
			0	0.002	0.0001	0.12	0.011	0	0.005
Prescribed Medication (binom)	1	nonsuperior	0.424	-0.147 **	0.369	-0.31 **	0.13 **	0.261 *	-0.386 **
			0.154	0.004	0.197	0	0.012	0.035	0.00001
Non-Prescribed Medication (binom)	1	nonsuperior	0.206 **	0.18 *	0.443	0.384 *	0.132 **	0.41	-0.131 **
			0.001	0.015	0.099	0.034	0.001	0.063	0.00001

Presenteeism weighted (gen poisson)	0.5	nonsuperior	0.138 **	0.103 *	0.011 **	-0.053 **	-0.007 **	-0.019 **	0.086 **
			0.002	0.036	0.0001	0.00002	0.001	0.0003	0.011

Supplement G: Outcome variables

Health outcomes:

Self-perceived health: using the EQ5D visual analog score: 0-100 (from worst health the employee can imagine to the best health the employee can imagine)

Incidence of musculoskeletal health functioning problems: Count of how many respondents indicated to have musculoskeletal health functioning problems (Nordic Musculoskeletal Questionnaire)

Stress: Score that represents the risk of stress using the Copenhagen Psychosocial Questionnaire: 0-12 (from low to high risk of stress)

Burnout: Score that represents the risk of burnout using the Copenhagen Psychosocial Questionnaire: 0-16 (from low to high risk of burnout)

Sleep problems: Score that represents the risk of sleep problems using the Copenhagen Psychosocial Questionnaire: 0-16 (from low to high risk of burnout)

General mental health: Score that represents the general mental health using the General Health Questionnaire: 0-12 (from worst to best mental health)

Need for recovery after: Score that represents the need for recovery after work, using the Need for Recovery scale: 0-11 (from lowest to highest need for recovery after work)

Role conflicts: Score that represents the occurrence of role conflicts or problems using the Copenhagen Psychosocial Questionnaire: 0-16 (from low to high risk of burnout)

Productivity outcomes:

Absenteeism: Absenteeism in days absent using the IMTA Productivity Cost Questionnaire (iPCQ)

Presenteeism: Presenteeism using the IMTA Productivity Cost Questionnaire (iPCQ), which uses a visual analog score to indicate how much work the employee could perform compared to a normal working day

Healthcare outcomes:

Spontaneous consultations with the occupational physician: Self-reported number of spontaneous consultations the employee had with the occupational physician

Referrals to other health providers: Number of contact moments with other healthcare providers based on a referral by the occupational physician

Work-related consultations with other healthcare providers: Self-reported number of work-related consultations the employee had with other healthcare providers

Prescribed medication: self-reported use of prescribed medication (no-yes)

Non-prescribed medication: self-reported use of non-prescribed medication (no-yes)

Secondary outcomes:

Health literacy: Score representing the health literacy of the employee, based on an adapted version of the European Health Literacy Survey (EU-HLS): 0-64 (low to high health literacy

Trust in Physician: Score representing the trust of the employee in his occupational physician, based on an adapted version of the Trust in Physician Scale: 0-55 (low to high trust)

Job Satisfaction: Score that represents job satisfaction, using the Copenhagen Psychosocial Questionnaire: 0-20 (from low to high job satisfaction)

Turnover intention: Score that represents turnover intention, using VBBA: 0-4 (from low to high turnover intention)

Worry about health: Score that represents worry about health, using custom questions on intensity 1-4 (how worried are you about your health), frequency 1-5 (how often do you worry about your health), and a combination of both questions 0-1.55
